# Intergenerational transmission of lockdown consequences: prognosis of the longer-run persistence of COVID-19 in Latin America

**DOI:** 10.1007/s10888-021-09501-x

**Published:** 2021-07-31

**Authors:** Guido Neidhöfer, Nora Lustig, Mariano Tommasi

**Affiliations:** 1 ZEW – Leibniz Centre for European Economic Research, Mannheim, Germany; 2 Commitment to Equity Institute, Tulane University, New Orleans, LA USA; 3 Center of Studies for Human Development, Universidad de San Andres, Victoria, Argentina

**Keywords:** COVID-19, Lockdowns, Human capital, School closures, Intergenerational persistence, Education, Inequality, Latin America, I24, I38, J62

## Abstract

The shock on human capital caused by COVID-19 is likely to have long lasting consequences, especially for children of low-educated families. Applying a counterfactual exercise we project the effects of school closures and other lockdown policies on the intergenerational persistence of education in 17 Latin American countries. First, we retrieve detailed information on school lockdowns and on the policies enacted to support education from home in each country. Then, we use these information to estimate the potential impact of the pandemic on schooling, high school completion, and intergenerational associations. In addition, we account for educational disruptions related to household income shocks. Our findings show that, despite that mitigation policies were able to partly reduce instructional losses in some countries, the educational attainment of the most vulnerable could be seriously affected. In particular, the likelihood of children from low educated families to attain a secondary schooling degree could fall substantially.

**Supplementary Information:**

The online version contains supplementary material available at 10.1007/s10888-021-09501-x.

**JEL codes** I24 · I38 · J62

✉ Guido Neidhöfer
guido.neidhoefer@zew.de

Nora Lustig
nlustig@tulane.edu

Mariano Tommasi
tommasi@udesa.edu.ar

### 1 Introduction

This paper is concerned with the long-run effects of the COVID-19 pandemic on the intergenerational persistence of inequality in Latin America. It focuses on one of the main drivers of these effects: namely, the closure of educational facilities established in most countries to limit the spread of the disease. Instructional time has a direct impact on students and its reduction has adverse effects on educational outcomes (Lavy 2015). School closures that occurred in several circumstances, for instance during the 1916 polio pandemic and World War II, had negative effects on the educational attainment of affected children (e.g. Ichino and Winter-Ebmer 2004; Meyers and Thomasson 2017). Usually, disadvantaged children are particularly exposed during school closures and suffer major losses (e.g. Jaume and Willén 2019; Alexander et al. 2007). This dynamic is further exacerbated by the economic shocks suffered by households during the COVID-19 pandemic (see e.g. Adams-Prassl et al. 2020; Blundell et al. 2020; Chetty et al. 2020; Lustig et al. 2020). School closures and lowered incomes are likely to decrease human capital investments for children living in poorer households in particular. These effects are likely to be irreversible and have negative consequences on earnings of the affected population throughout their lives (e.g. Almond 2006). Depending on how pervasive and irreversible the impact of school closures on the entire population in school is, the negative impact of the pandemic on intergenerational inequality and equality of opportunity could be persistent and even last for several generations. The strengthening of the correlation between children and their parents' education due to the "schooling shock" plays a major role in this process.

Intergenerational associations of educational achievements are an insightful measure for the persistence between generations of the distribution of resources in a society, with a strong correlation between parents' and children's education pointing at low equality of opportunity (Hertz et al. 2007; Narayan et al. 2018; Neidhöfer et al. 2018). Hence, our aim is to estimate the potential long-lasting effects of COVID-19, simulating the extent to which the pandemic is intensifying the intergenerational persistence of education and deteriorating high school completion rates among individuals with different parental background. The impact on children's human capital is quantified taking into account the amount of instructional time lost due to the COVID-19 pandemic (see e.g. Abadzi 2009; Adda, 2016). We extend this approach to consider several variables that show considerable variation across Latin American countries: closure and reopening of educational facilities; online and offline interventions aimed at facilitating learning at home; the distribution of internet coverage among socio-economic groups; epidemiological parameters affecting the likelihood of infection and death of household members; household income losses; and, social assistance measures designed to mitigate the pandemic-related income losses. Furthermore, our analysis takes into account that parents have different capabilities to substitute formal schooling. High-educated parents may be able to compensate the instructional loss fully, while children of low-educated parents mostly rely on the supply of schooling provided by the education systems (in class or through the support of home learning).

Recent studies analyzed the impact of school closures on learning outcomes, either with surveys and real time data (e.g. Angrist et al. 2020; Aucejo et al. 2020; Chetty et al. 2020), standardized test scores (e.g. Engzell et al. 2021; Maldonado and De Witte 2020), or simulating the potential aggregate impact (e.g. Azevedo et al. 2020; Hanushek and Woessmann 2020; Jang and Yum 2020) and its consequences for long-run earnings and welfare (e.g. Fuchs-Schündeln et al. 2020; Psacharopoulos et al. 2020). In all current analyses, the heterogeneity in the learning losses experienced by students of different socioeconomic background due to COVID-19 is largely documented. Aucejo et al. (2020) find that lower-income students in the US are 55% more likely than their higher-income peers to have delayed graduation in higher education. Andrew et al. (2020) shows that in the UK during the school lockdown primary and secondary school children from households in the highest income quintile learned around 1.5 and 1 h more at home, respectively, than their peers from the poorer income quintiles. Bacher-Hicks et al. (2021) show that in the US internet searches for online learning resources are disproportionately high in geographic areas with higher average income and better internet access. The findings of Chetty et al. (2020) about the learning progress recorded on an online math platform during the school lockdown confirm this picture as well: while children in high income areas recovered quickly to the pre-crisis levels, children in lower-income areas remained persistently under the baseline level. Test score data for Belgium and the Netherlands shows increasing patterns in inequality within and between schools, and stronger learning losses in schools with a larger share of disadvantaged students from less-educated households (Engzell et al. 2021; Maldonado and De Witte 2020). To the best of our knowledge, none of them investigated changes in intergenerational associations due to COVID-19 or differences across countries regarding education policies during the lockdown.

Our study estimates the impact of the pandemic on the accumulation and allocation of human capital, and projects, for the first time to the best of our knowledge, its consequences for intergenerational persistence of education and equality of educational opportunities in Latin America, one of the most affected regions worldwide in terms of COVID-19 deaths and economic costs (IMF 2020; ECLAC 2020). In November 2020, around eight months after the beginning of the pandemic, 97% of children in the region were still out of classrooms (UNICEF 2020). Acevedo et al. (2020) estimate that due to the situation educational exclusion in the region could increase by 15%. To estimate the effects on intergenerational persistence, we proceed in three steps. First, using data on parents and children's education from Latinobarometro, a representative survey including 18 Latin American countries, and the standard methods to measure intergenerational persistence, we estimate the slope coefficient, correlation coefficient, and rank correlation for the most recent pre-pandemic cohort available (i.e. individuals born between 1987 and 1994) extending the analysis of Neidhöfer et al. (2018).[1] Second, taking into account school closures and eventual re-openings, several indicators of offline and online learning, as well as other relevant characteristics, we simulate the heterogeneous impact by family background of COVID-19 school closures and other shocks on school achievements of these individuals. This step generates a counterfactual scenario. Third, we proceed to re-estimate the slope coefficient, correlation coefficient, and rank correlation for the counterfactual "post-pandemic" scenario. Additionally, we estimate the high-school completion rates for children of low- and high-educated parents for the observed and the counterfactual scenario and proceed to compare the two.

To the best of our knowledge, this study is the first to quantify and compare the long-term implications of the pandemic on educational attainment and intergenerational mobility in a unified framework for a large set of countries. Our findings show that, despite that educational mitigation policies were able to partly reduce learning losses in some countries, the pandemic puts at risk the educational attainments of the most vulnerable and equality of opportunity. The likelihood of children from low educated families to complete high-school could fall by 20 percentage points or

[1] Intergenerational mobility estimates from Neidhöfer et al. (2018) are available on http://mobilitylatam.website .

more, reversing decades of progress made in Latin America in terms of access to education among children from disadvantaged households, and the average slope coefficient of intergenerational education persistence could rise by 7% from a regional average of 0.36 to 0.39.

## 2 Counterfactual estimation exercise

### 2.1 Intergenerational persistence

Human capital investments may be affected by demand- and supply-side factors that limit or enhance the investment opportunities of families. The COVID-19 pandemic affects both. School closures affect supply, and falling household incomes and illness affect demand for education. Assuming a human capital production function where the primary production factors are schooling and the family, the main component that drives the uneven shock of the pandemic, and its intergenerational persistence, is the learning loss suffered by children.

An established way to display the process of intergenerational persistence is through the following equation:

$$Y_0 = \beta Y_{-1} + \varepsilon. \tag{1}$$

Here, $Y$ is a measure of human capital or socioeconomic status, for instance education measured in years of schooling, for two subsequent generations within a family. $\varepsilon$ is the error term. Hence, it is implied that the process of intergenerational persistence is autoregressive of the first order and the slope coefficient $\beta$ measures the velocity of regression to the population mean (Becker and Tomes 1979, 1986). $\beta$ is also the parameter that shows the persistence of advantages and disadvantages transmitted from parents to children. It can be empirically estimated through a linear regression of children's outcomes on the characteristics of their parents. Higher values of $\beta$ indicate a higher level of association between parents' and children's outcomes (for example their educational attainments) and, thus, lower equality of opportunity (see Hertz et al., 2008). For instance, using years of schooling as outcome measure, a value for $\beta$ of 0.5 says that an advantage of one year of schooling of one family over other families in the same generation is associated with a transmission of half of this advantage to the next generation. A $\beta$ equal to 1 means that there is no churning of the advantages, i.e. there is zero mobility over time, and a $\beta$ equal to zero means that children's educational attainments are independent of the attainment of their parents, i.e. there is perfect mobility.

Our aim is to quantify the potential effect of school closures and lockdown measures caused by the COVID-19 pandemic on the intergenerational association of education in Latin America. For this purpose, we first estimate $\beta$ for the years of schooling of the cohort of young individuals that already left the education system in pre-COVID-19 times but is closest to the cohort of children that are now in the education system. Then, we simulate the shock of the pandemic on the human capital of these individuals to obtain a counterfactual measure of their predicted years of schooling. Finally, we re-estimate the intergenerational persistence on this simulated counterfactual outcome and compare the estimated parameters.[2] The exercise reveals how the intergenerational persistence of

[2] In our main application, we estimate the slope coefficient $\beta$. To test the robustness of our results, we also estimate the correlation coefficient and rank correlation. Performing the exercise with either of these measures yields qualitatively the same results. Hence, we report only the estimates of the slope coefficient in the main text and the other two measures in the Supplemental Material.

this cohort would have changed if these individuals had suffered a human capital loss equivalent to the one potentially caused in 2020 by COVID-19.

To provide a further, more intuitive measure of educational persistence and absolute upward mobility, we estimate the probability of individuals with different parental educational background to achieve a certain level of education. Following Neidhöfer et al. (2018) we predict the average probability of individuals with low and high-educated parents to achieve at least a secondary education degree. For comparability across countries and cohorts, we define the two types of parental education as follows: low parental education, i.e. less than a secondary education degree; and high parental education, i.e. at least secondary education. Again, these measures are estimated with the actual education of individuals as reported in the data, as well as after the counterfactual simulation of the COVID-19-shock.

### 2.2 Post-pandemic human capital

The main driver of the human capital shock suffered by children due to the COVID-19 pandemic has been the closure of schools aimed at limiting the spread of the disease. Several studies have shown that reduced instructional time lowers academic achievement (e.g. Jaume and Willén 2019). As evidenced by studies measuring the educational gaps after summer vacations, it is likely that this situation mostly affects children from disadvantaged backgrounds (e.g. Alexander et al. 2007). These children have fewer educational opportunities beyond school, while their parents are less prepared to support them in the educational process at home. Furthermore, the capabilities of families to facilitate learning from home are uneven. They depend on the abilities of parents, their acknowledgement of the value of education for lifetime outcomes, the available educational tools and resources, and the availability of computers and internet coverage. Most parents with higher education can help their children with learning from home. They might be able to replace teachers and possibly even improve their children's skills due to the one–to-one interaction.[3] Hence, children living in low-educated families have clear disadvantages. Indeed, real time data on learning during the COVID-19 pandemic confirms this disproportion in educational efforts, outcomes, and expectations between poor and rich families (e.g. Aucejo et al. 2020; Bacher-Hicks et al. 2021; Chetty et al. 2020; Engzell et al. 2021; Maldonado and De Witte 2020). However, the impact of school closures on children's human capital also depends on the effort done by the countries to provide online and offline home learning resources. Hence, in our exercise to simulate the educational loss caused by the pandemic we take into account all of these aspects: school closures, educational mitigation strategies, and the ability of parents to replace formal schooling in educational facilities.

Our simulation exercise builds on measuring the amount of instructional time lost due to the closure of schools (see e.g. Abadzi 2009; Adda, 2016). Conceptually, education is translated into an equivalent measure of human capital, where one year of schooling corresponds to an increase in human capital by one unit. Hence, the instructional time lost due the pandemic, measured as share of the regular school year, is taken into account as human capital loss and subtracted from the years of schooling of the individual. What results is a counterfactual measure of the individual's educational attainments if she would have suffered the loss of instructional time caused by the pandemic. We extend the approach to take into account

[3] Evidence for very young children shows that spending most of the time with their high skilled parents, rather than in childcare, significantly increases their cognitive abilities in the long run (Fort et al. 2020).

impacts on individuals in different countries and taking into account their parents' level of education. Hereby, we choose the assumptions of the model as conservative as possible, such that the resulting human capital loss should constitute a lower bound of the potential deficit.[4] For instance, we assume that in periods of school closures students do not forget what they had learned before, despite that this might be the case as shown for the summer vacation period (e.g. Cooper et al. 1996). Later, in Section 4.6, we discuss the caveats and potential limitations of this approach and their implications for the interpretation of our results.

The conceptual basis of the exercise is a human capital production function defined by three components: schooling, family environment, and innate abilities (e.g. Hanushek and Woessmann 2012). We assume innate abilities not to be affected by the pandemic and concentrate on the role played by schooling and family factors. The post-pandemic counterfactual education $\widehat{e_{ijc}}$ of individual $i$ living in country $c$ whose parents have level of education $j$ is defined as her actually measured education $e_{ijc}$ from which the $\kappa$ share of the year of school lost due to COVID-19 is subtracted.

$$\widehat{e_{ijc}} = e_{ijc} - \kappa_{ijc} \tag{2}$$

Conceptually, Eq. (2) measures how strong the pandemic-related shock reduces the education of the individual; ranging from zero, i.e. the education of the individual is not affected at all by the pandemic, to one, which stands for an entire year of schooling lost. This reduction varies by country and parental educational background.

$\kappa$ has two interacting components, which are directly retrieved from the theoretical framework of the human capital production function described before:

$$\kappa_{ijc} = K_{ijc} \cdot \alpha_{ij} \tag{3}$$

$K$ is the share of instructional time lost and $\alpha$ stands for one minus the parental factor of substitution. Both parameters can range from zero to one. $K = 0$ means schools remain open for the entire year or mitigation strategies are able to substitute in-person classes perfectly, while $K = 1$ means schools are closed for the entire year and no educational mitigation strategies are enacted. $\alpha = 0$ corresponds to the case in which all parents of educational background $j$ are able to perfectly replace in-school attendance and $\alpha = 1$ to the case in which not a single parent of educational background $j$ is able to do it. We will first describe the calculation of $K$ and below the one of $\alpha$.

$K$ includes the days lost due to school closures and is compensated by the effort of countries to support the education of children when not at school, as well as structural characteristics of the country's internet connectivity and digital infrastructure that have been proven useful to learn from home. Furthermore, it encompasses the health risk suffered by the individual and her family due to the spread and mortality rate of COVID-19 in the country of residence. To set these parameters, we draw from various data sources. Formally, $K$ is:

[4] This aspect is challenged by the potential existence of differential age- or grade-effects and discussed in Section 4.6. It particularly applies to the estimates of the slope, correlation and rank correlation coefficients that consider the entire distribution of years of education. Instead, changes in the likelihood of high-school completion are driven by individuals at the margin (those that completed secondary education but did not follow up with tertiary education) and, hence, not directly affected by this potential problem. Therefore, in case of the likelihood of high-school completion by parental background we can more safely assume our estimates to constitute a lower bound. Furthermore, we perform robustness checks to account for grade-effects.

$$K_{ijc} = \frac{t_c\left(1 - f_c \cdot \delta - n_c \cdot P\left(A_{ijc} = 1\right) \cdot (1 - \delta)\right) + \tau_{ic}}{T_c}. \tag{4}$$

Here, $t$ is the amount of days of school lost from the closure of schools to the day of reopening, subtracting the country-varying days of school vacation lying in between, and $T$ the amount of school days in a regular year of schooling.[5] The term enclosed in the first parenthesis measures the compensation of schooling facilitated by public investments in home learning tools. $f$ and $n$ are indices that we construct to capture the extensiveness of offline and online education tools during the pandemic.[6] $\delta$ is a weight between the two set of resources that defines their relative effectivity. In the main analysis we set this weight to 0.5, meaning that ceteris paribus both offline and online learning resources are equally capable to transmit learning material and may together be able to replace a regular day in class. The index of online education is further interacted with the probability of the individual to have access to internet $A$, which we approximate by the internet coverage among people in socioeconomic group $j$ in the country. While for all individuals the school closure is compensated by the offline learning resources provided by their country's education system, the $A_{jc}$ share of individuals whose parents have education level $j$ in country $c$ are assumed to have access to internet and, hence, to online learning resources, as well. This step of the analysis enters the simulation as follows: First, we estimate the distribution of internet coverage based on household surveys for each country (the methodology is explained in Table S3 in the Supplemental Material). Then, we randomly draw from our sample the $A$ share of individuals whose parents have education level $j$ in country $c$. Finally, for these individuals, we subtract from the total days of schooling lost ($t_c - t_c f_c \delta$) the days compensated with online learning resources ($n_c(1-\delta) \cdot t_c$), while for the remaining $(1-A)$ share of the population within group $j$ the instructional loss is only compensated by offline learning.

$\tau$ captures the instructional loss due to health shocks suffered by households (see e.g. Aucejo et al. 2020). It includes two components:

$$\tau_{ic} = \tau^q \cdot P(q_{ic} = 1) + \tau^d \cdot P(d_{ic} = 1). \tag{5}$$

$q$ is the infection of one of the household members with COVID-19 and $d$ death due to the latter. To estimate the probabilities of these events to occur, the number of COVID-19 infections and deaths per inhabitant in the country is multiplied by the average country-level household size. $\tau^q$ and $\tau^d$ are the respective amounts of days of schooling lost due to the occurrence of the two events, either due to the time the child has to stay at home in case someone in the household is infected, or due to a reduction of home learning caused by the overall burden associated to infection, illness or even death of a family member. We set $\tau^q$ to

[5] For those countries where schools have not been reopened yet the date is set to the beginning of November 2020.

[6] Governments made different efforts for children to keep on studying during the pandemic. Mainly, they gave out printed copies, sent educational material via cellphone, and broadcasted educational contents through radio and TV. The index $f$ was calculated as the share of tools used among the four mentioned. On top of these efforts governments also provided resources for online learning. Using information on digital platforms, virtual tutoring, digital resources, and digital content repositories we constructed the index $n$, which captures the use of these tools by the country's educational system. $f$ ($n$) is one if all the offline (online) educational tools were used by the country's education system during the school closure, and zero if none of them was used. For all other combinations the indexes lie between these values. More detailed information on these measures and the sources of information are included in the Supplemental Material (Table S3).

the average days of symptom duration, which has been found to be around one week (i.e. five days of schooling), and $\tau^d$ to a three week loss of instructional time (i.e. 15 days).[7] Again, these losses are attributed randomly to the within-group share of the sample that mirrors the probability of infection and death in the family.

The parental factor of substitution captures the capabilities of parents to substitute formal schooling. Through Eq. (3) it defines the strength in which the educational loss defined by $K$ is suffered by children, depending on the circumstances they face in terms of parental background. We define $\alpha$, which is one minus the parental factor of substitution, as

$$\alpha_j = 1 - \frac{e_j^p}{\max\left(e_j^p\right)}. \tag{6}$$

$e_j^p$ are the completed years of schooling of parents with educational degree $j$. The range of years of schooling goes hereby from zero years, equivalent to illiterate parents, to 15 for parents with completed tertiary education. Consequently, the extreme values of $\alpha$ are zero for high educated parents that may fully substitute the educational losses, and one for children of illiterate parents who completely absorb the educational shock caused by the pandemic. For other levels of parental education the value of $\alpha$ lies within this interval. Note that $\alpha$ may be the actual capacity of parents to help their children with the learning material, or more broadly the informational advantage about the value of education for lifetime success and the connected investments to support it, such as parental time, the availability of technological devices at home, private schooling, and tutoring. Generally, $\alpha$ captures the higher propensity of parents with higher education and socio-economic status to invest in their children's education (see e.g. Heckman and Mosso 2014). Since $\alpha$ is interacted with the whole term $K$ in Eq. (3) this interaction eventually defines the strength of the individual loss in instructional time due to school closure, taking also into account the differential exposure to health risks for households of different socio-economic status during the COVID-19 pandemic (see e.g. Blundell et al. 2020).

Although our calibration of the parental factor of substitution does not directly derive from current empirical estimates of the effect of school closures on learning losses by parental socioeconomic background, it meets the overall pattern observed in empirical studies. For instance, Jaume and Willén (2019) find that the negative effect of teacher strikes on educational outcomes in Argentina was strong for children of low-educated parents and mostly zero for parents with high education. Maldonado and De Witte (2020) show that after the school closures caused by the COVID-19 pandemic in Belgium the learning losses were high in schools where most children have low educated mothers and nil in schools with a high share of high-educated mothers. Engzell et al. (2021) find a substantial difference in the test score gap

[7] It has been shown that death of a parent might cause serious educational losses and even school dropout (e.g. Case and Ardington 2006; Gertler et al. 2004). However, since the likelihood of death due to COVID-19 rises with age and existing medical preconditions, we mainly assume older household members to be affected rather than parents of younger children and adolescents, and choose a shorter instructional loss due to the death of a household member. Anyway, because of the rather low probability of infection and death these assumptions do not affect our estimates significantly.

between children of high and low-educated parents caused by the school closure in the Netherlands.[8]

The parameter $\alpha$ can be understood in either of the two following ways: as the proportional loss of $K$ experienced universally by all children of parents with a $j$ level of education, or as a certain share of children of parents with a $j$ level of education who lose the entire $K$ proportion of the school year, while the rest lose none. We call the first scenario *concentrated losses* and the latter *dispersed losses.*[9] Conceptually, in the dispersed scenario $\alpha$ is the degree in which all parents with educational degree $j$ are able to substitute schooling, while in the concentrated scenario, it is the likelihood of parents with educational degree $j$ to be a perfect substitute of schooling. While in the former the shock is distributed to the degree $\alpha \cdot K$ evenly to all individuals with the respective parental background, the latter attributes a shock of the amount of $K$ to a randomly selected $\alpha$-share of the population within those groups; i.e. $\alpha_{ij} = \alpha_j$ for all $i$ with parental education $j$ in the dispersed scenario, and $\alpha_{ij} = 1$ with probability $\alpha_j$ and $\alpha_{ij} = 0$ with probability $(1 - \alpha_j)$ in the concentrated scenario. For example, for children whose parents complete primary education only, we assumed $\alpha = 1 - 0.33$ (parental factor of substitution = 5/15). In the dispersed scenario, this means that all the children whose parents completed only primary school lose 2/3 times $K$ of the school year. In the concentrated scenario, this should be interpreted that 2/3 of the children in this group lose the entire $K$ amount of the year, while the remaining 1/3 the children in this group complete the year normally.

We report the simulated concentrated educational losses in the main body of the text and the dispersed losses in Fig. S2 in the Supplemental Material. Note that the slope coefficient of the regression is going to be very similar for either scenario because the average loss for each group where parents have a $j$ level of education is almost the same in both cases.[10] In other words, the coefficient is insensitive to something that is important: the dispersion within the losers. That is why it is crucial to assess the impact on an indicator which is sensitive such as the high school completion rate.[11] We will discuss the drawbacks and limitations of this additional measure of educational persistence when describing the results in Section 4.2 and verify the consistency of the measure to changing the main assumptions of the model with robustness checks in Section 4.6.

[8] The fact that Engzell et al. (2021) find a significant negative effect also for children of high educated parents depends on the broad definition of this category that mirrors the classification used by the Dutch Ministry of Education to determine school funding, namely “at least one parent with a degree above lower secondary education”. Indeed, our calibration allows an educational gap to exist for children of parents whose level of education is below a completed tertiary degree.

[9] This distinction mimics the existence of two scenarios of *dispersed* and *concentrated* losses contemplated, for instance, in Lustig et al. (2020), in considering the effects of COVID-19 on incomes.

[10] When one does not consider the likelihood of internet access, of infection, and death in the analysis – which in both simulated scenarios, the dispersed and the concentrated one, are modelled as probabilities and randomly attributed to the $A$, $q$ *or* $d$ share of individuals with parental education $j$ in country $c$ – the expected value of $\kappa_{ijc}$, as well as average years of education, are equivalent in both scenarios. This applies also to the special case that these probabilities are zero or one for all $i$ with parental education $j$. For each probability between zero and one the expected relative instructional loss $\kappa$ in the dispersed scenario is defined by the interaction between the value of $\alpha$, the probability to have access to internet, and the probability of infection and death in the household. In the concentrated scenario instead, it is defined by the conditional probability of all events to occur simultaneously. For instance, to suffer an instructional loss with probability $1 - \alpha_j$ and to have no access to internet with probability $1 - A_{jc}$.

[11] The rank correlation in this case is sensitive too, but with a mechanical component: the rank correlation will be lower in the concentrated losses case, because the fact that all individuals with parental education $j$ loose the same amount of the school year leaves the number of ranks unchanged. Conversely, if only some loose, while others do not, the number of ranks is higher than in the status quo.

### 3 Data

The micro-data we use to perform the simulations and estimate the potential effects of the estimated instructional loss on schooling and intergenerational persistence of education derives from Latinobarometro, a representative survey including 18 Latin American countries.[12] Latinobarometro is particularly suitable for a multi-country analysis of intergenerational persistence of education because it includes detailed and harmonized information about the education of individuals and retrospective information about the education of their parent with the highest educational degree (see Neidhöfer 2019). Estimates of educational attainment and its distribution deriving from Latinobarometro are highly comparable to estimates obtained with national household surveys (Neidhöfer et al. 2018). We use the survey waves from 1998 to 2017 and restrict the sample to individuals born between 1987 and 1994 who were at least 23 years old when responding to the survey. While the age limit ensures that the individuals should have completed their education when responding to the survey, we select the cohort 1987–1994 to warrant that our simulations in terms of mobility are as close as possible to the potential mobility of the cohort of children and youth currently in school in 2020. The further back we would go, the weaker would this assumption be, given that the region has experienced a remarkable level of educational upgrading. Our final sample comprises 10,524 individuals evenly distributed across countries.

To reduce measurement error, rather than using the information on actual years of schooling included in the survey for both individuals and their parents, we impute the regular years of education associated with the respective educational degree.[13] In what follows, we report our estimates obtained by weighting for the inverse probability of selection, normalizing the weights over the different survey waves, and without inclusion of control variables. Estimates obtained controlling for sex and survey year fixed effects differ only slightly and not significantly.

To compute the single components of $K$, we retrieve the information on school closures, educational policies, and other structural characteristics of the countries from different sources including national education ministries, international organizations, and macro data sources (see Table S3 in the Supplemental Material).

### 4 Results

#### 4.1 Instructional loss due to school lockdowns

Using the procedure described in Section 2.2, we simulate the instructional loss for each individual in each country and socioeconomic group $j$. Table 1 shows the values of all

[12] In this analysis, we include all Latin American countries included in the survey with the exemption of Nicaragua because the country never officially closed schools during the pandemic. Generally, there have been concerns about the overall handling of the pandemic in Nicaragua (see Mather et al. 2020, and subsequent replies on this article).

[13] In the main analysis, years of schooling are imputed based on the following scheme (in parenthesis the imputed years of schooling): Illiterate (0), incomplete primary (3), complete primary (5), incomplete secondary (8), complete secondary (12), incomplete tertiary (13), complete tertiary (15). We perform robustness checks replacing the values for incomplete degrees with the country-specific modal years of schooling in the respective educational categories and find no significant differences to the main estimates.

**Table 1** Indicators used to compute the instructional loss

| | *Schooling* | | | | *Connectivity among socioeconomic groups by the education of the household head* | | | | | | *COVID-19 (09/20)* | | |
|---|---|---|---|---|---|---|---|---|---|---|---|---|---|
| *c* | *t* | *T* | *f* | *n* | *less than primary* | *complete primary* | *incomplete secondary* | *complete secondary* | *incomplete tertiary* | *complete tertiary* | *Cases per inh.* | *Deaths per inh.* | *Avg. hh size* |
| ARG | 154 | 180 | 0.75 | 0.69 | 0.63 | 0.67 | 0.69 | 0.72 | 0.78 | 0.81 | 0.01090 | 0.00023 | 3.3 |
| BOL | 157 | 200 | 0.50 | 0.25 | 0.12 | 0.28 | 0.26 | 0.44 | 0.76 | 1.00 | 0.01062 | 0.00062 | 3.5 |
| BRA | 157 | 200 | 0.50 | 0.63 | 0.49 | 0.59 | 0.68 | 0.84 | 0.91 | 0.92 | 0.01972 | 0.00060 | 3.3 |
| CHL | 154 | 190 | 0.75 | 0.75 | 0.68 | 0.67 | 0.77 | 0.84 | 0.90 | 0.92 | 0.02245 | 0.00062 | 3.6 |
| COL | 150 | 200 | 0.75 | 0.75 | 0.32 | 0.48 | 0.66 | 1.00 | 1.00 | 1.00 | 0.01350 | 0.00043 | 3.5 |
| CRI | 154 | 200 | 0.50 | 0.50 | 0.66 | 0.73 | 0.76 | 0.77 | 0.79 | 0.79 | 0.00989 | 0.00011 | 3.5 |
| DOM | 72 | 197 | 0.25 | 0.44 | 0.67 | 0.74 | 0.77 | 0.78 | 0.80 | 0.80 | 0.00932 | 0.00018 | 3.5 |
| ECU | 100 | 200 | 0.75 | 0.56 | 0.29 | 0.44 | 0.51 | 0.69 | 0.85 | 0.99 | 0.00637 | 0.00061 | 3.8 |
| SLV | 158 | 200 | 0.50 | 0.38 | 0.14 | 0.25 | 0.31 | 0.55 | 0.96 | 1.00 | 0.00411 | 0.00012 | 4.1 |
| GTM | 145 | 180 | 0.75 | 0.56 | 0.31 | 0.47 | 0.68 | 1.00 | 1.00 | 1.00 | 0.00448 | 0.00016 | 4.8 |
| HND | 162 | 200 | 1.00 | 0.56 | 0.13 | 0.24 | 0.29 | 0.51 | 0.90 | 0.94 | 0.00669 | 0.00021 | 3.9 |
| MEX | 136 | 185 | 0.25 | 0.50 | 0.33 | 0.48 | 0.65 | 0.93 | 1.00 | 1.00 | 0.00504 | 0.00054 | 3.7 |
| PAN | 163 | 185 | 0.50 | 0.38 | 0.30 | 0.44 | 0.51 | 0.69 | 0.86 | 1.00 | 0.02317 | 0.00050 | 3.7 |
| PRY | 158 | 200 | 0.75 | 0.38 | 0.28 | 0.45 | 0.68 | 1.00 | 1.00 | 1.00 | 0.00344 | 0.00007 | 4.6 |
| PER | 154 | 185 | 0.50 | 0.69 | 0.15 | 0.27 | 0.37 | 0.61 | 0.87 | 1.00 | 0.02141 | 0.00093 | 3.8 |
| URY | 75 | 180 | 0.25 | 1.00 | 0.46 | 0.55 | 0.70 | 0.82 | 0.90 | 1.00 | 0.00049 | 0.00001 | 2.8 |
| VEN | 121 | 180 | 0.25 | 0.33 | 0.63 | 0.66 | 0.69 | 0.72 | 0.78 | 0.80 | 0.00191 | 0.00002 | 3.3 |

t are the days of instructional lost (assuming schools reopen in November 2020 if they are still closed), T the days in a regular year of schooling, f and n indices that measure the alternative supply of education during school closures through offline (TV, radio, cellphone, printed copies) and online (internet) learning provided by the education system. Reported COVID-19 cases and deaths per inhabitant recorded in September 2020. Sources: see Table S3 in the Supplemental Material variables used to compute $K$ for each Latin American country. A detailed explanation on how all these variables are computed and the underlying sources can be found in Table S3 in the Supplemental Material. Table 2 shows the values of $\kappa$, resulting from the interaction between schooling features, captured by $K$ and $\alpha$. The loss in instructional time associated to these values can be interpreted as the average share of the school year lost by individuals in the respective socio-economic group.

We observe that, while for individuals in the higher parental background classes (at least completed secondary education) the instructional loss is rather low, namely lower than or close to 10% in all countries, substantial differences between countries exist among individuals with low educational background. Bolivia, El Salvador, Mexico, Panama, and Peru are the countries with the highest average estimated instructional losses, around 60% of the school year; while in Ecuador, Dominican Republic, and Uruguay the lowest educational impact is recorded. These differences depend in part on the reopening of schools, but are also clearly marked by the effort of education systems to provide alternative learning tools and the pre-existent digital infrastructure to provide access to online resources. The health risk associated with COVID-19 only marginally contributes to the instructional loss.

### 4.2 Secondary education completion

Although the simulation framework that we develop mainly works through changes in the years of schooling and their distribution, for illustrational purposes we also focus on secondary education completion. First, to offer a further intuitive baseline result about the potential impact of the pandemic on educational attainment and inequality. Second, because of the particular policy relevance of this specific cutoff. Increasing secondary school completion rates

**Table 2** Average share of instructional loss by parental background

| $c$ | $E[\kappa_{jc}]$ | | | | | | |
|---|---|---|---|---|---|---|---|
| | *j*= *illiterate* | *incomplete primary* | *complete primary* | *incomplete secondary* | *complete secondary* | *incomplete tertiary* | *complete tertiary* |
| ARG | 0.35 | 0.28 | 0.23 | 0.16 | 0.06 | 0.04 | 0.00 |
| BOL | 0.58 | 0.46 | 0.37 | 0.26 | 0.11 | 0.07 | 0.00 |
| BRA | 0.47 | 0.38 | 0.30 | 0.20 | 0.08 | 0.05 | 0.00 |
| CHL | 0.30 | 0.24 | 0.20 | 0.13 | 0.05 | 0.03 | 0.00 |
| COL | 0.38 | 0.30 | 0.22 | 0.13 | 0.04 | 0.03 | 0.00 |
| CRI | 0.45 | 0.36 | 0.29 | 0.20 | 0.09 | 0.06 | 0.00 |
| DOM | 0.27 | 0.21 | 0.17 | 0.12 | 0.05 | 0.03 | 0.00 |
| ECU | 0.27 | 0.22 | 0.17 | 0.11 | 0.04 | 0.03 | 0.00 |
| SLV | 0.57 | 0.46 | 0.37 | 0.26 | 0.10 | 0.06 | 0.00 |
| GTM | 0.43 | 0.35 | 0.27 | 0.16 | 0.06 | 0.04 | 0.00 |
| HND | 0.38 | 0.30 | 0.23 | 0.16 | 0.06 | 0.03 | 0.00 |
| MEX | 0.58 | 0.47 | 0.37 | 0.24 | 0.09 | 0.06 | 0.00 |
| PAN | 0.61 | 0.49 | 0.39 | 0.27 | 0.11 | 0.07 | 0.00 |
| PRY | 0.45 | 0.36 | 0.29 | 0.18 | 0.07 | 0.05 | 0.00 |
| PER | 0.58 | 0.47 | 0.37 | 0.24 | 0.09 | 0.05 | 0.00 |
| URY | 0.27 | 0.22 | 0.17 | 0.10 | 0.04 | 0.02 | 0.00 |
| VEN | 0.52 | 0.41 | 0.34 | 0.24 | 0.10 | 0.07 | 0.00 |

Numbers indicate the average share of the year of schooling lost due to COVID-19 for each socio-economic group $j$ (by parental educational background). Source: Own estimates based on various sources; see Table 1 and Table S3 in the Supplemental Material are an indicator of the educational expansions that characterized Latin America for the past decades (Levy and Schady 2013). Especially children from low-educated families benefited from this expansions, leading to a substantial decrease of intergenerational persistence over time (Neidhöfer et al. 2018). Furthermore, changes in inequality in the region have been associated with changes in returns just above and below high school completion (López-Calva and Lustig 2010).

Figure 1 shows the overall share of individuals who attain a secondary educational degree (i.e. attain at least 12 years of schooling) in the sample for each country before and after consideration of the COVID-19 shock, using the estimates for the instructional loss reported in Table 2. The share for the regular school year is estimated using the actual years of schooling of the individuals, while the second bar shows the estimate using the counterfactual adjusted years of schooling instead, i.e. $\widehat{e_{ijc}}$ from Eq. (2). On average, the likelihood to complete secondary education in Latin America drops from 56% to 42%. There is, however, quite a bit of variation across countries. The country with the sharpest decline is Brazil, where we observe a decrease of 23 percentage points. The one with the lowest decline is Uruguay with a 6 percentage point decrease. While the generally lower pre-pandemic baseline high school completion rate in Uruguay in comparison to Brazil surely explains part of this difference, it is also remarkable that Uruguay is one of the countries in our sample with the lowest projected average instructional loss across socioeconomic levels (see Table 2). Particularly, Uruguay stands out because of the shorter duration of the school closure, as well as the availability of online resources to mitigate learning losses (see Table 1).

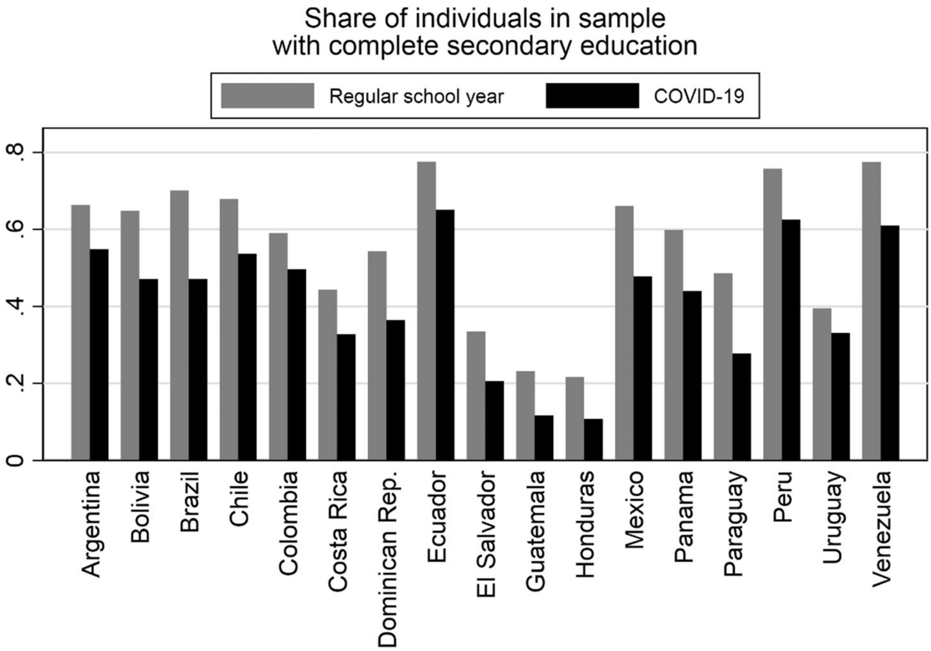

**Fig. 1** Weighted share of individuals in sample who complete secondary education before and after imputation of the COVID-19 shock on human capital. Notes: Completed secondary education is equivalent to 12 full years of schooling. First scenario shows actual share of individuals in sample with completed secondary schooling. Second scenario shows estimates of the same share after simulation of the COVID-19 shock. Source: Latinobarometro, own estimates

In Fig. 2 we report the likelihood to complete a secondary education degree for individuals with low and highly educated parents (corresponding to parents with and without a completed secondary school). This likelihood is estimated by the predicted probability to attain 12 or more years of schooling.[14] Again, this likelihood is estimated for the baseline scenario and after simulation of the COVID-19 shock, i.e. after subtraction of $\kappa_{jc}$. Altogether, these results highlight the heterogeneous impact of the pandemic on educational attainment associated to parental background. While the likelihood of individuals from high-educated families to complete high school is hardly affected, this same likelihood for individuals with low-educated parents is considerably lower in the post-pandemic scenario with respect to the baseline. On average, the likelihood of completing high school for children of low-educated families declines by almost 20 percentage points.[15] Again, the sharpest decline is recorded in Brazil (32 percentage points) and the lowest in Uruguay (9 percentage points). In Guatemala in Honduras the probability of secondary school completion of individuals from low-educated families even falls under 10%. We also estimate high-school completion rates by parental background for men and women separately and find similar patterns among both groups. Results are reported in Fig. S4 in the Supplemental Material.

The differences we find in the impact of the pandemic on the high-school completion rates of individuals with low- and high-educated parents depend on the distribution of education just around the cut-off (i.e. secondary school completion, which is equivalent to twelve years of schooling). Indeed, changes to high school completion rates in this part of the exercise respond to changes at the extensive margin (i.e. the share of children loosing at least one day of schooling due to the pandemic) rather than to changes at the intensive margin (i.e. the amount of days of schooling lost due to the pandemic). Since the impact of the pandemic on this measure of educational persistence is driven by individuals who completed secondary schooling and not more, what is crucial for the results is tertiary education enrollment. While most children of high-educated parents continue their educational career spending at least some years in tertiary education, many children of low-educated families that experience educational upward mobility attain at most a secondary degree, and hence fall under the threshold of 12 years of schooling after simulation of the COVID-shock.[16]

Figure 3 shows the trend in the average degree of absolute educational upward mobility (i.e. the likelihood of secondary school completion for individuals whose parents did not complete

[14] Indeed, studies confirm that absence in school leads to significant negative effects on achievements, which could eventually lead to school dropout (e.g. Kubitschek et al. 2005). However, we also relax this assumption taking into account that moderate instructional losses might be recovered and that education systems might allow for extensions to complete the school year. The results of these additional applications is discussed in Section 4.6.

[15] We choose completed secondary education as threshold to define high and low-educated parents mainly to warrant comparability with past educational mobility estimates (e.g. Neidhöfer et al. 2018). Although we observe ample cross-country heterogeneity, in Latin America parents with at least a completed secondary degree constitute about the top 33% of the distribution, while in all countries this group is larger than 10% (see Table S4 in the Supplemental Material). If we define high-educated parents as parents with at least completed primary school, and low-educated parents as parents with an educational level below that threshold, the average decline in the likelihood of secondary school completion of children from low-educated families in Latin America is almost the same. Figure S6 in the Supplemental Material shows the estimated likelihoods applying this alternative threshold.

[16] On average for the 1987–94 cohort in Latin America, 37% of individuals with low-educated parents and a completed secondary degree continue their educational career afterwards (less than 16% of all individuals with low-educated parents have at least some years of tertiary education). Among children of high-educated parents, this statistic is 65% (53% of all individuals with high-educated parents). See also the transition matrix in Table S5 and, for a complete picture for each country, Table S6 in the Supplemental Material.

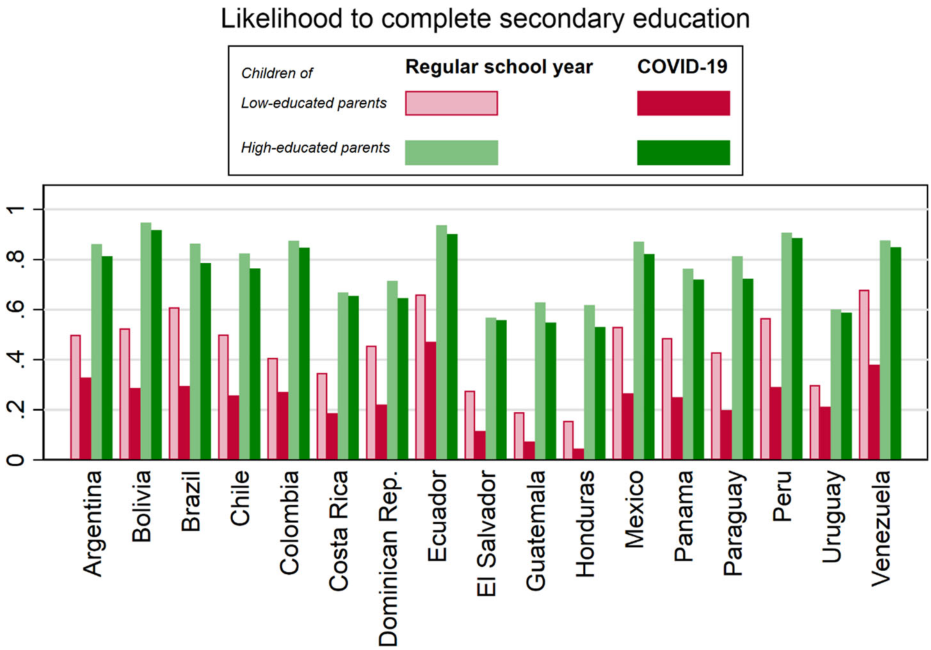

**Fig. 2** Estimated likelihood to complete secondary education by socioeconomic background before and after imputation of the COVID-19 shock. Notes: Bars show the likelihood to complete at least 12 years of schooling before and after simulation of the COVID-19 shock on education. High educated parents have at least completed secondary education, low educated parents less than completed secondary education. Source: Latinobarometro, own estimates high school) for the region, including a projection for the cohorts closest to high school completion in 2020, namely the 2001–2005 birth cohort.[17] This cohort is the oldest one directly affected by the COVID-19 crisis while at the same time these children are potentially enrolled in the education system and close to high school graduation. We report the expected degree of educational upward mobility of this cohort with and without the impact of the COVID-19 shock (i.e. a decrease of the likelihood by 20 percentage points due to the pandemic as shown by our simulation exercise). As can be seen in the Figure such a decrease in high school graduation rates of disadvantaged children would bring the region several decades back. The resulting rather low average degree of educational upward mobility was lastly reported in Latin America for cohorts born in the 1960s (see also Neidhöfer et al. 2018).

#### 4.3 Effect on intergenerational persistence

Figure 4 shows the estimated degree of intergenerational persistence of the sample – measured by the slope coefficient – for the baseline (i.e. reported years of education) and after simulation of the counterfactual background specific learning loss due to the pandemic (see Tables 1 and 2), as well as their difference. A hypothetical worst case scenario is included as further benchmark in the graph. The worst-case scenario computes the learning loss assuming that

[17] The likelihood of the two youngest cohorts, for which data is not available yet, has been obtained by linear extrapolation of the series from 1941 to 1995.

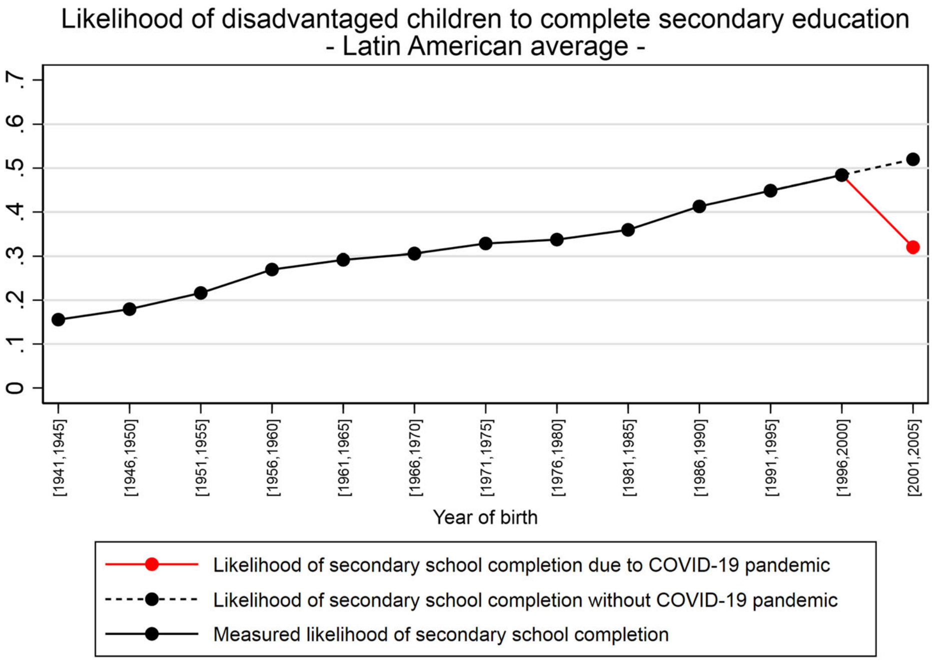

**Fig. 3** Updated trend in educational upward mobility. Notes: Dots show the likelihood of children whose parents have less than secondary education to complete secondary education. Unweighted average. Source: Latinobarometro, own estimates schools remain closed for an entire year and no mitigation strategies to reduce the learning loss are enacted; i.e. $K = 1$.[18]

We observe that, although the enacted policies prove effective to reduce the potential learning loss due to school closures, intergenerational persistence rises in all countries. The slope coefficient in the post-pandemic counterfactual is, on average for all Latin American countries, around 7% higher than the baseline. To put this figure into context, between 1940 and 1990 – a period marked by upward educational mobility – the slope coefficient for Latin America, measured as an average over all countries, declined by 4% from one four-year birth cohort to the next (own calculations based on the estimates provided in Neidhöfer et al. 2018). In other words, the loss in intergenerational mobility could be significant. The strongest differences are observed in Peru (0.39 vs. 0.43), Mexico (0.30 vs. 0.34), and Bolivia (0.40 vs. 0.44), and the weakest differences in Honduras (0.56 vs. 0.57), the country with the highest slope coefficient in the baseline Table 3 shows the estimated differences and their corresponding standard errors.

#### 4.4 Extension: drop-out due to household income loss

We extend the exercise to account for additional shocks that might affect human capital investments among families. Hence, this part of the exercise takes into account changes in

[18] Table S1 in the Supplemental Material shows the corresponding values and the difference between the actually measured degree of persistence and the counterfactual scenarios.

**Table 3** Estimates and standard errors

| | Average years of education | | | Slope coefficient | | | | Absolute upward mobility | | | | Top persistence | | | |
|---|---|---|---|---|---|---|---|---|---|---|---|---|---|---|---|
| | Regular | w/COVID | -Δ% | Regular | w/COVID | Diff. | s.e. | Regular | w/COVID | Diff. | s.e. | Regular | w/COVID | Diff. | s.e. |
| Argentina | 10.89 | 10.81 | 0.7 | 0.362 | 0.386 | −0.024 | 0.002 | 0.499 | 0.329 | 0.170 | 0.018 | 0.861 | 0.813 | 0.047 | 0.012 |
| Bolivia | 10.26 | 10.11 | 1.5 | 0.400 | 0.435 | −0.036 | 0.001 | 0.525 | 0.287 | 0.239 | 0.018 | 0.946 | 0.916 | 0.030 | 0.012 |
| Brazil | 10.37 | 10.27 | 1.0 | 0.272 | 0.303 | −0.031 | 0.002 | 0.610 | 0.294 | 0.316 | 0.026 | 0.863 | 0.787 | 0.076 | 0.019 |
| Chile | 10.90 | 10.83 | 0.7 | 0.349 | 0.372 | −0.023 | 0.007 | 0.500 | 0.257 | 0.243 | 0.055 | 0.824 | 0.765 | 0.059 | 0.015 |
| Colombia | 10.40 | 10.29 | 1.1 | 0.494 | 0.523 | −0.029 | 0.001 | 0.407 | 0.272 | 0.135 | 0.013 | 0.874 | 0.847 | 0.027 | 0.008 |
| Costa Rica | 8.76 | 8.61 | 1.7 | 0.316 | 0.342 | −0.025 | 0.002 | 0.347 | 0.187 | 0.160 | 0.019 | 0.669 | 0.655 | 0.014 | 0.010 |
| Dominican Rep. | 8.87 | 8.82 | 0.6 | 0.273 | 0.287 | −0.014 | 0.001 | 0.456 | 0.220 | 0.236 | 0.021 | 0.715 | 0.646 | 0.068 | 0.019 |
| Ecuador | 11.39 | 11.35 | 0.3 | 0.318 | 0.336 | −0.018 | 0.001 | 0.661 | 0.472 | 0.189 | 0.019 | 0.936 | 0.901 | 0.035 | 0.011 |
| El Salvador | 6.66 | 6.42 | 3.7 | 0.414 | 0.441 | −0.027 | 0.002 | 0.276 | 0.116 | 0.160 | 0.020 | 0.568 | 0.558 | 0.010 | 0.010 |
| Guatemala | 5.59 | 5.39 | 3.5 | 0.480 | 0.496 | −0.016 | 0.002 | 0.191 | 0.074 | 0.117 | 0.016 | 0.629 | 0.548 | 0.080 | 0.040 |
| Honduras | 5.38 | 5.22 | 3.1 | 0.560 | 0.570 | −0.010 | 0.002 | 0.156 | 0.046 | 0.111 | 0.014 | 0.618 | 0.530 | 0.088 | 0.034 |
| Mexico | 10.37 | 10.23 | 1.4 | 0.302 | 0.339 | −0.037 | 0.001 | 0.531 | 0.265 | 0.266 | 0.026 | 0.870 | 0.823 | 0.048 | 0.014 |
| Panama | 10.07 | 9.96 | 1.1 | 0.327 | 0.360 | −0.033 | 0.002 | 0.487 | 0.250 | 0.237 | 0.025 | 0.764 | 0.720 | 0.044 | 0.016 |
| Paraguay | 8.17 | 7.97 | 2.3 | 0.394 | 0.427 | −0.033 | 0.002 | 0.429 | 0.200 | 0.230 | 0.020 | 0.813 | 0.724 | 0.089 | 0.030 |
| Peru | 11.19 | 11.10 | 0.8 | 0.389 | 0.426 | −0.038 | 0.001 | 0.567 | 0.291 | 0.276 | 0.027 | 0.906 | 0.885 | 0.021 | 0.007 |
| Uruguay | 9.56 | 9.47 | 1.0 | 0.356 | 0.375 | −0.019 | 0.002 | 0.299 | 0.212 | 0.087 | 0.015 | 0.601 | 0.588 | 0.013 | 0.009 |
| Venezuela | 11.76 | 11.70 | 0.5 | 0.181 | 0.215 | −0.034 | 0.002 | 0.679 | 0.380 | 0.299 | 0.028 | 0.875 | 0.848 | 0.027 | 0.008 |

Table shows average years of education and intergenerational mobility estimates for the cohort 1987–1994. ‘Regular’ shows the actually measured level of intergenerational persistence. w/COVID shows the post-pandemic counterfactual after simulation of the COVID-19 shock. Diff. shows the difference between the regular school year and the counterfactual, and s.e. its corresponding bootstrapped standard error obtained with 100 replications. -Δ% shows the years of schooling decrease in percentage. Slope coefficient is the coefficient of a linear regression of children’s years of education on the years of education of their parents. Absolute upward mobility is the likelihood of children with low-educated parents to complete high school. Top persistence is the same likelihood for children with high-educated parents. Source: Latinobarometro, own estimates

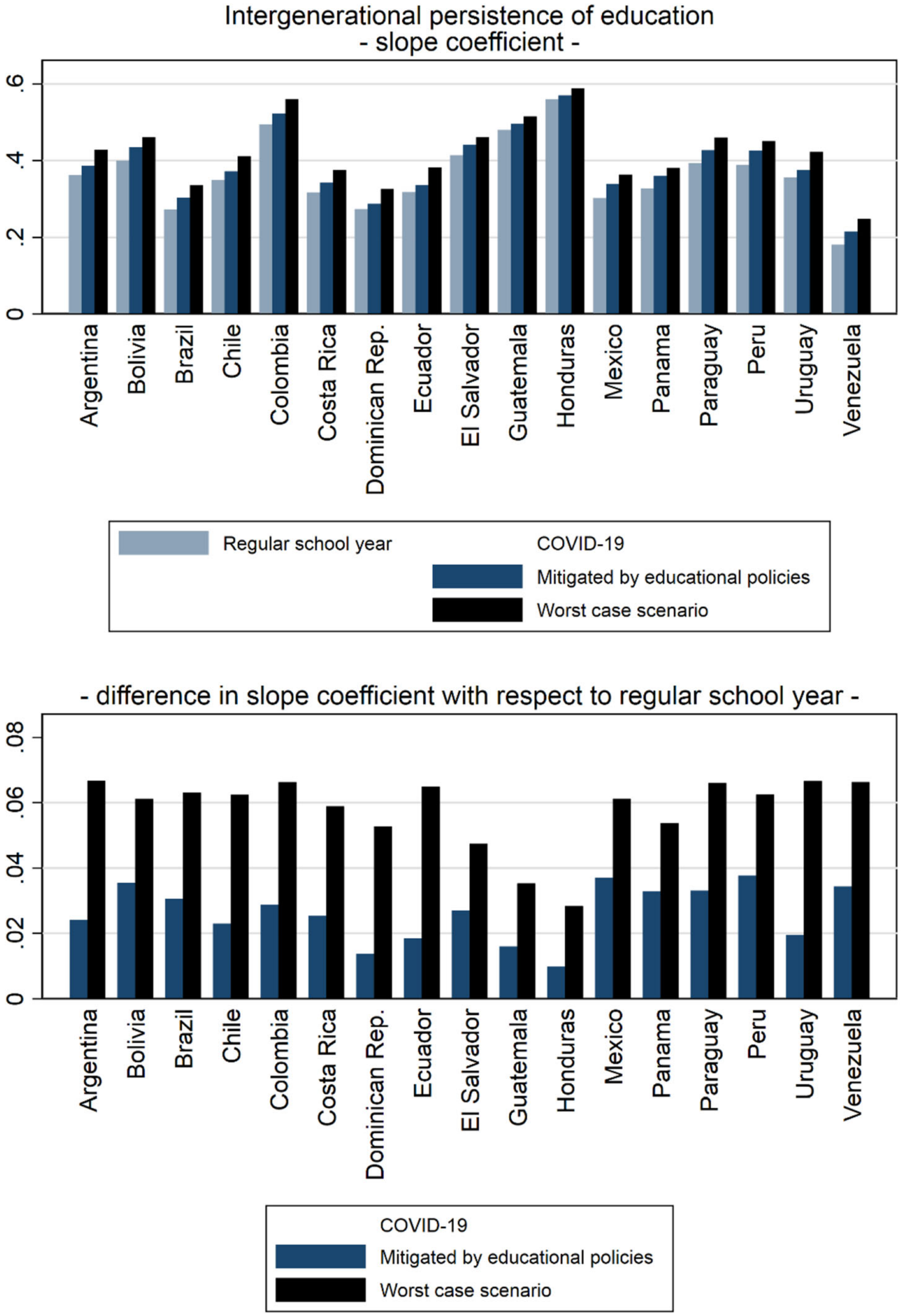

**Fig. 4** Intergenerational persistence of education in Latin America before and after imputation of the COVID-19 shock on human capital. Notes: Worst case scenario shows an instructional loss equivalent to 100% of the school year without any compensatory effect of mitigation policies. Source: Latinobarometro, own estimates demand side factors that affect the education of children and interact with the factors mainly related to the supply of education analyzed so far. As has been shown, parental job loss and household income shocks may cause educational drop out (see e.g. Duryea et al. 2007; Cerutti et al. 2019; Thomas et al. 2004). However, if the income shock depends on an economic crisis, declining opportunity costs of leaving school to enter the labor force may also lead to higher educational enrollment (e.g. Ferreira and Schady 2009; Torche 2010). Hence, the resulting overall effect on educational attainment is ambiguous and may vary by family background characteristics. To take into account that the likelihood of drop out may depend on parental socioeconomic background we perform the exercise setting the probability of educational drop out, defined by a loss of an entire year of schooling, to $\alpha$. This estimate yields an upper bound of the effect of income loss on education, especially at the bottom of the distribution. This upper bound is useful to evaluate the qualitative significance deriving from the additional impact of household income shocks, on top of the effects studied in the sections before. The new counterfactual measure of years of schooling is

$$\widetilde{e_{ijc}} = \widehat{e_{ijc}} - \alpha_j \cdot D_{jc} \tag{7}$$

where $\widehat{e_{ijc}}$ are the counterfactual years of schooling defined in Eq. (2), $\alpha$ the inverse of the parental factor of substitution from Eq. (6), and $D_{jc}$ the probability of parents with educational background $j$ in country $c$ to suffer an important income shock due to the pandemic. To estimate these probabilities, we rely on the data and microsimulation exercise adopted in Lustig et al. (2020) to Argentina, Brazil, Colombia, and Mexico. We define $D_{jc}$ as a loss of more than 50% of income, and simulate the probability of households to lose this amount of income due to the COVID-19 pandemic for each level of education of the household head. The simulated probabilities for two scenarios, namely with and without inclusion of the economic mitigation strategies enacted by the countries to cushion income losses, are shown in Table S2 in the Supplemental Material.[19] The strongest income losses are registered in the middle of the distribution, where the greatest proportion of household income is at risk, and lower at the top and at the bottom.

Figure 5 shows the resulting changes in intergenerational persistence and educational upward mobility. Besides of the already reported increase in persistence related to school closures and health risks, we observe a small additional increase as a consequence of parental job loss. This increase is absorbed, in part but not entirely, by the enacted mitigation strategies. The same picture emerges analyzing the likelihood of individuals with low parental background to attain a secondary schooling degree. In this case, for all countries but Argentina the additional (quite small) impact of parental job loss on the probability to drop out from school is not compensated by the enacted economic mitigation strategies.[20]

In conclusion, this extension of the exercise highlights that the impact of the COVID-19 pandemic on education and intergenerational persistence is mainly driven by the closure of schools, the cushioning effect of educational mitigation strategies, and infrastructural characteristics, as the distribution of internet coverage among the population. These results confirm

[19] For an exact description of the economic mitigation strategies enacted by countries, as well as the methodology and data sources to obtain these estimates, see Lustig et al. (2020).

[20] As shown by Ciaschi (2020) while the probability of school dropout of male children raises due to parental job loss, the probability of female children might be not affected. To simplify the exercise, we do not condition on gender. However, because of the low additional effect of parental job loss beyond supply side factors related to school closures, we do not expect this to significantly affect our estimates.

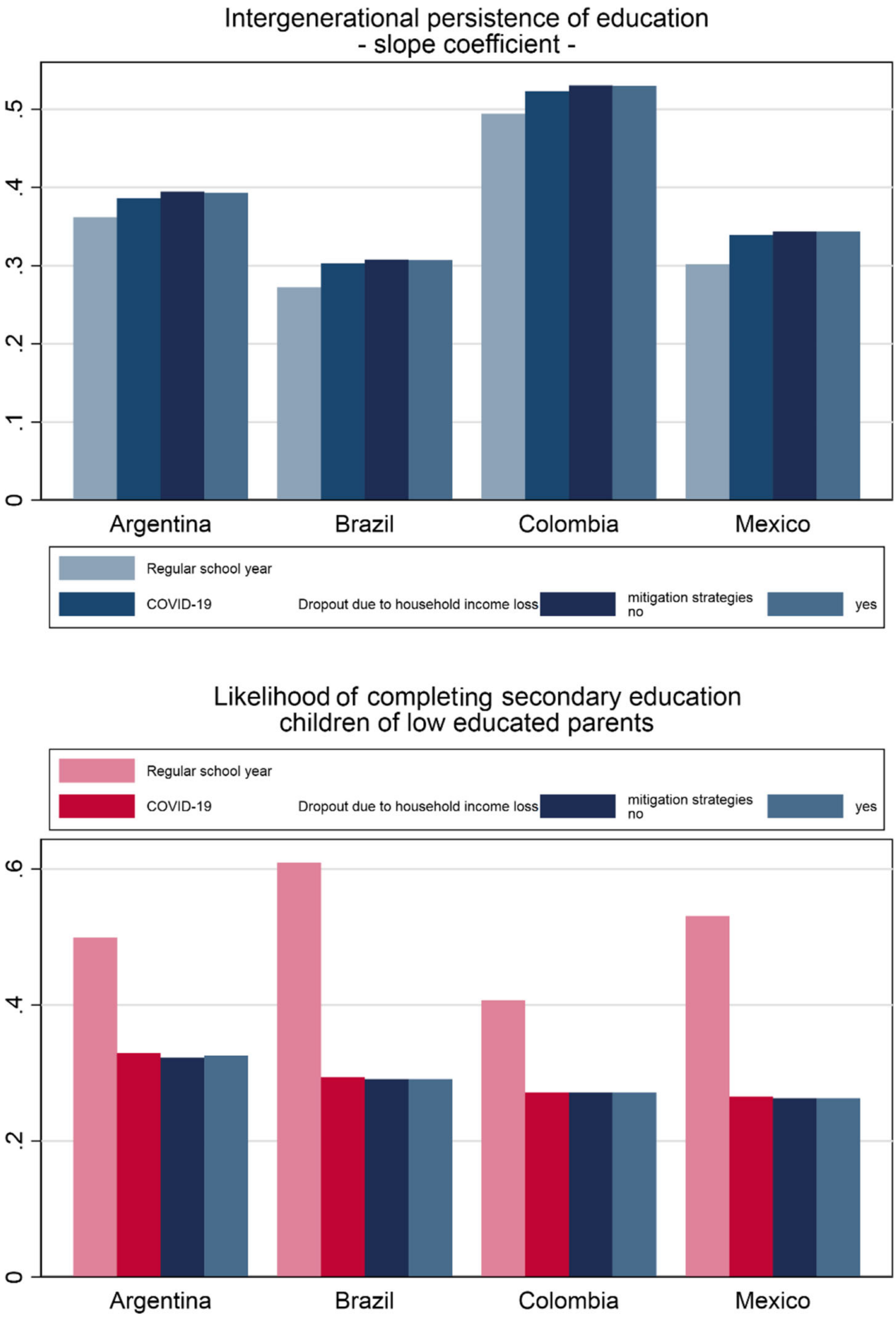

**Fig. 5** Intergenerational persistence of education accounting for the additional effect of household income losses due to the pandemic. Notes: First counterfactual scenario takes into account the instructional loss due to school closures and health shocks caused by COVID-19. Two additional counterfactual scenarios account for the likelihood of educational drop out due to household income losses caused by the pandemics: firstly, without considering economic mitigation strategies; secondly, considering the cushioning effect of economic mitigation strategies (see Lustig et al. 2020). Source: Latinobarometro, own estimates the findings of Fuchs-Schündeln et al. (2020) whose model predicts that the negative impact of the COVID-19 shock on children's long-run welfare is driven by school closures, while the income shocks suffered by households during this crisis play a secondary role. Policies mainly supporting the demand for education with cash transfers, which result to be effective to improve education of vulnerable children in regular times (e.g. Fiszbein and Schady 2009; Molina Millán et al. 2019; Neidhöfer and Niño-Zarazúa 2019), are hardly effective in a context of closed educational facilities.

#### 4.5 Mitigation through online learning

The adoption and further diffusion of educational technologies and online learning tools has been both employed and exhaustively discussed as a measure to reduce instructional losses during the COVID-19 pandemic (e.g. Clark et al. 2020). Our aim in this last part of the exercise is to evaluate whether improved online learning in the context of Latin American countries may reduce the unequal effect of school closures and support equality of opportunity. Figure 6 shows the correlation between the country-level extensiveness of online learning and overall internet coverage with predicted average instructional losses during the pandemic.[21] On a first sight there seems to be a positive association. Hence, we analyze if major dedication and concentration to online learning tools can close the learning gap.

We simulate an increase in the extensiveness of online learning tools to the maximum; i.e. we set $n = 1$ in Eq. (4) which means that the quality of online learning is such to substitute in-class schooling perfectly. Furthermore, we fix the dedication of the educational effort in times of school closures at 100% to online tools; i.e. $\delta = 0$ in Eq. (4).

Figure 7 shows, in the upper graph, the estimated likelihood of children from low educated background to complete secondary education in the scenario with improved online learning, and compares it to the two other scenarios discussed in Section 4.2. We observe that, despite improving online learning increases secondary school completion rates, it is not enough to close the gap caused by the pandemic. Indeed, in countries with very low internet coverage among lower socioeconomic groups the change in completion rates is almost non-existent. The lower graph in Fig. 7 shows the implications of this for intergenerational persistence: For countries with very unequal internet coverage, a complete concentration on online education and its contemporaneous improvement would cause even higher educational persistence. Hence, although possibly more cost-effective and efficient than the production of offline education tools, relying completely on online learning in times of school closures is not equalizing. In contrast, given the current distribution of access to internet in many Latin American countries it might even increase educational inequality. This should be even exacerbated by the lower availability of computers and other digital devices among poor households, which we are not taking into account in this analysis. Important and targeted investments in digital infrastructure and internet connectivity are necessary for online learning to equalize the playing field. Alternatively, recent evidence shows that low-technology interventions such as SMS and direct phone calls can be effective in reducing learning gaps in a context of developing countries (Angrist et al. 2020).

[21] For a more detailed description of the indicators for online learning and internet coverage, see Table 1 and S3 in the Supplemental Material.

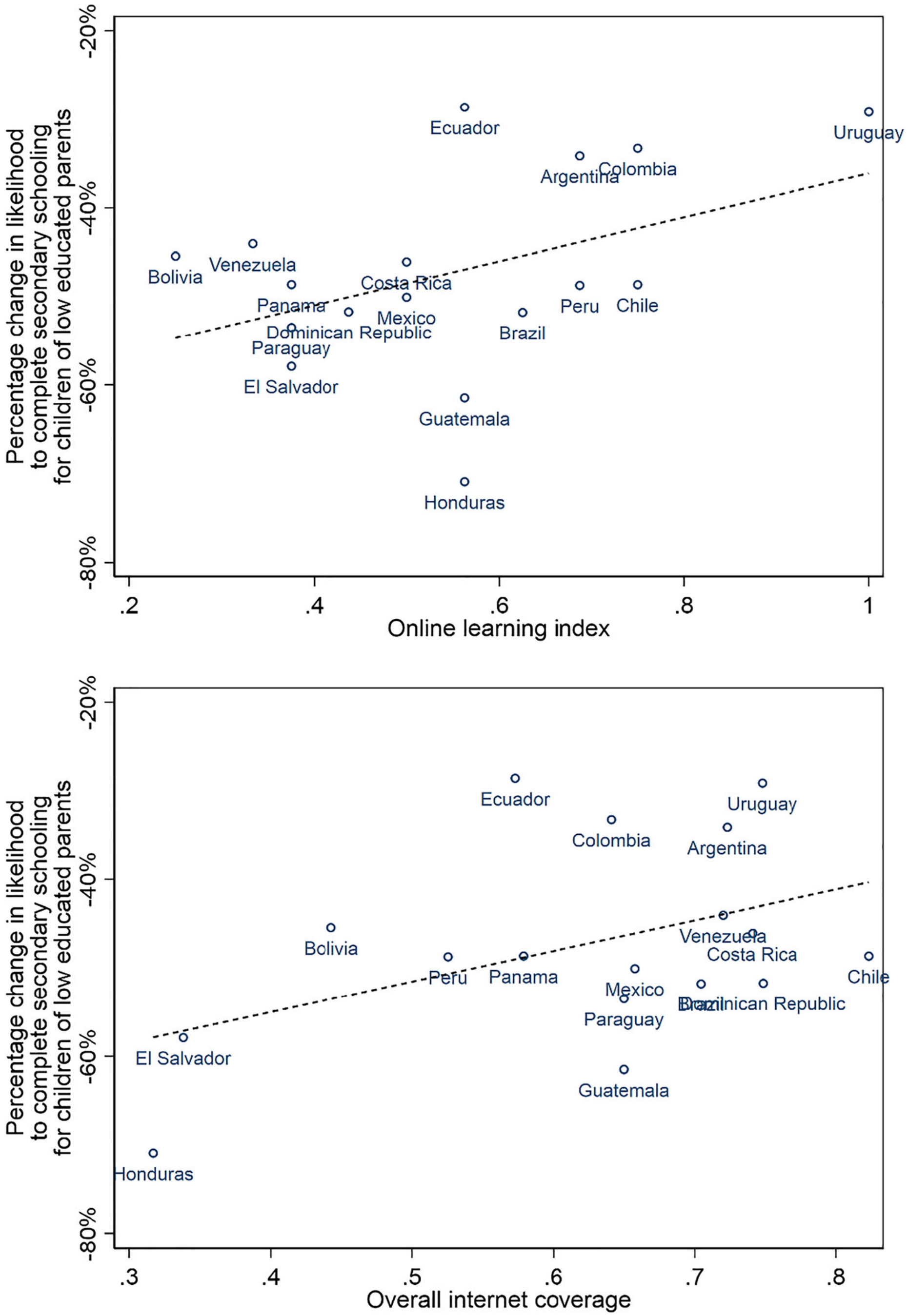

**Fig. 6** Online learning, internet coverage and average instructional loss. Notes: Graph shows the relationship between the average difference in intergenerational persistence at the bottom of the distribution (between the situation without COVID-19 and the post-pandemic counterfactual) and online learning. Online learning index computed based on distinct information, see Supplemental Material. Overall internet coverage in the country from World Bank data

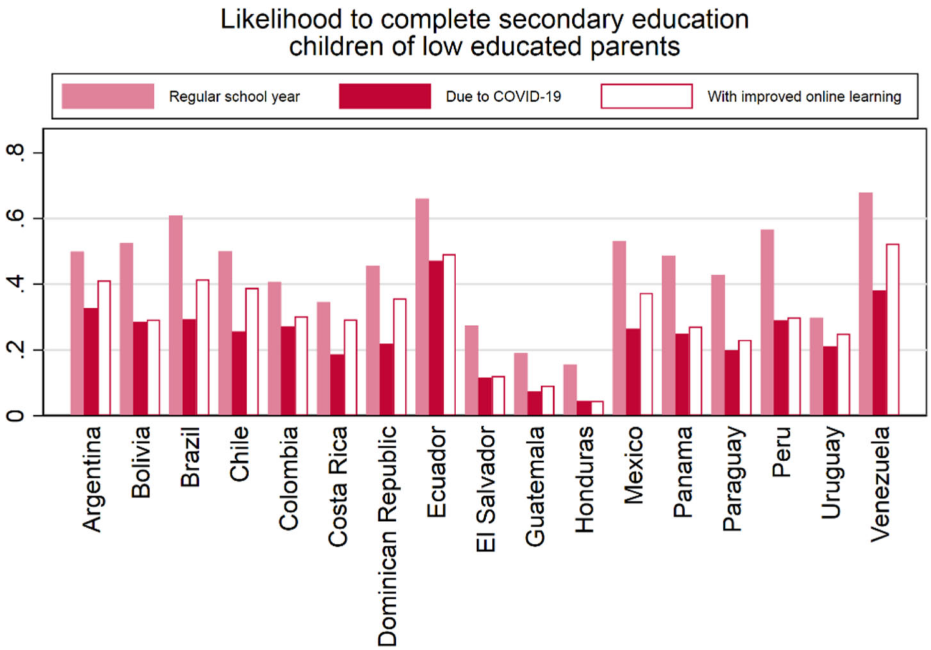

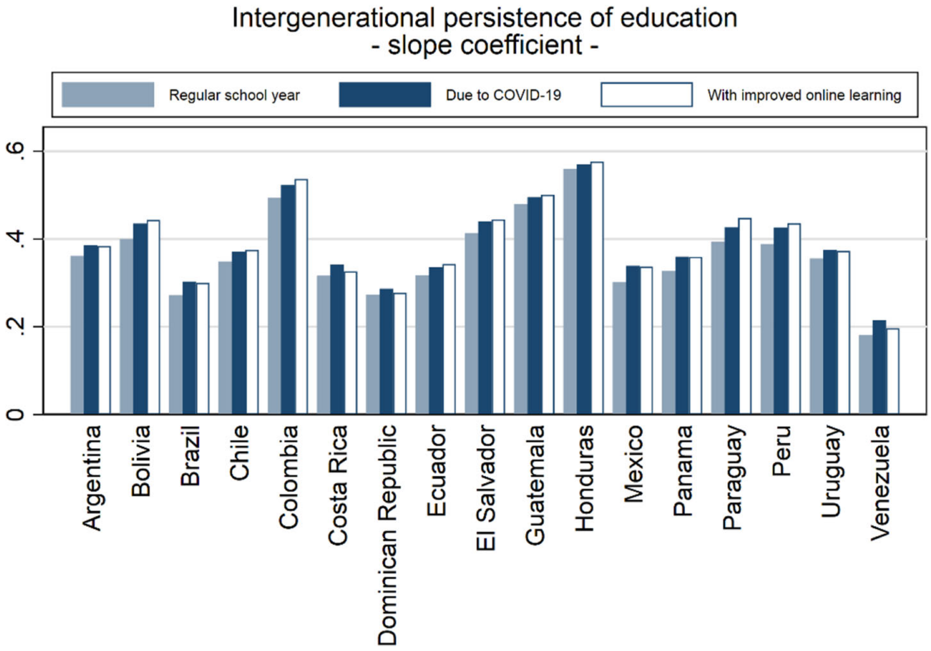

**Fig. 7** Simulated effect of improved online learning on intergenerational persistence. Notes: Simulated raise in the extensiveness of online learning tools to the maximum; i.e. $n = 1$ in Eq. (4) which means that the quality of online learning is such to substitute in-class schooling perfectly. Here, we fix the dedication of the educational effort in times of school closures at 100% to online tools; i.e. $\delta = 0$ in Eq. (4). Source: Latinobarometro, own estimates

#### 4.6 Robustness and limitations

To test the robustness of our results we relax two of the main assumptions of the model. First, as mentioned before, we perform a sensitivity analysis to estimate the impact of the pandemic in a scenario of *dispersed*, rather than *concentrated*, learning losses (see Fig. S2 in the Supplemental Material). In this application, secondary school completion is substantially lower. In almost all countries the predicted probability of low background children to complete secondary education reaches even levels close or above 20%. The results of this additional robustness check can be regarded as an upper bound estimate of the negative effect of the COVID-19 crisis.

Secondly, we relax the assumption that 12 full years of education are necessary to complete a secondary school degree. Albeit absence in school due to individual reasons indeed leads to significant negative effects on achievements, a moderate instructional loss shared by many students in the same school may not cause the same dramatic and unrecoverable educational loss (e.g. Kubitschek et al. 2005). Furthermore, in case of moderate instructional losses the education systems in certain countries might enable extensions for pupils to complete the school year (see e.g. UNESCO et al. 2020). Therefore, we estimate the likelihoods of secondary school completion setting different thresholds for the post-pandemic counterfactual: 11.75 and 11.5 years. These applications provide a more conservative estimate than the strict threshold of 12 years of schooling. In these cases, the assumption is that children will complete secondary education despite of the pandemic if their reported education in absence of the shock is a completed secondary degree or higher, and the suffered instructional loss is not higher than 25% or 50% of the school year. We observe that in these applications the gap in the completion of secondary schooling with respect to the regular year without COVID-19 is lower, and in few countries it may even vanish. However, even in the less stringent threshold level there are still remarkable differences with respect to the baseline in 11 of the 17 countries in the region. These results are shown in Fig. S3 in the Supplemental Material.

Still, our analysis has some caveats behind the intrinsic assumptions of the counterfactual exercise. First, we assume that individuals do not suffer a cumulative effect due to the learning loss, possibly leading to earlier educational drop out, but continue their educational trajectories as soon as regular schooling is re-established. Second, we do not directly consider the additional effect of school closures, and other situations connected to the pandemic, on externalities and other features, such as nutrition, obesity, mental health, teenage pregnancy, non-cognitive skills etc. (Wang et al. 2020). Surely, these other factors are crucial for human development, and shocks in these dimensions may decrease the upward mobility of vulnerable children as well (see e.g. Ferreira and Schady 2009; Almond et al. 2018). However, considering these aspects would clearly contribute to even stronger learning losses causing our current estimates to be a lower bound.

Third, in our evaluation the effects of the instructional loss are assumed to be the same for all ages and in all school grades or, equivalently, that all individuals in the sample are hit at the same age (or in the same grade). Conversely, the direction of the bias deriving from this assumption is a priori not clear. Possibly, it may be easier to make up for instructional losses in earlier grades, while harder later on, when the learning material is more intense. At the same time, older children might be more able to study on their own and depend less on their parents. Indeed, recent analyses of the long-term effects of school closures caused by COVID-19 come to contrasting conclusions: while the model by Fuchs-Schündeln et al. (2020) predicts that younger children are hurt more, Jang and Yum (2020) find that school closures reduce intergenerational mobility especially among older children. Anyway, especially for children from disadvantaged background it is unlikely that age-related effects offset the entire impact of school closures, especially since changes in the likelihood of high-school completion are driven by individuals at the margin (those that completed secondary education but did not follow up with tertiary education). However, the issue could apply particularly to the estimates of the slope, correlation and rank correlation coefficients that consider the entire distribution of years of education. Under consideration of potential age-related effects our estimates for these measures of persistence would constitute an upper rather than a lower bound of the effect of COVID-19. Hence, we perform robustness checks to account for grade-effects in the estimation of the slope, correlation and rank correlation coefficient. First, we assume that the effect for those with less than secondary education is nil (hence assuming that at earlier ages the instructional loss can be recovered, or that the pandemic shock hit in a year when these individuals already left the education system). Then, we estimate the reverse, namely that only those with secondary or more are affected by the instructional loss (which is similar to the scenario analyzed by the likelihood of high-school completion). In both cases the difference in coefficients between the baseline and the post-pandemic counterfactual are lower than in the main analysis (5% and 3%, respectively), but still sizeable in most of the countries.[22]

Fourth, our analysis exploits the variation in education policies across countries. Yet, what could also affect differences in instruction time reduction across children from different socio-economic backgrounds is a potentially differential implementation of policies across schools and districts, leading to (spatial) variation within countries. Insofar as these differential implementations are correlated with parental background, for instance because of differences between private and public schools or residential segregation, their effect is captured by the parental factor of substitution. Other sources of (geographical) heterogeneity in learning losses are a very interesting subject for future investigations.

Lastly, our simulations only encompass the human capital shock due to the COVID-19 pandemic, while assuming all other years to be regular and corresponding to a unitary increase in human capital. While this assumption is standard in the literature on the intergenerational persistence of education, we acknowledge that, particularly in developing countries, accumulated years of education of individuals are mostly a rather imprecise measure of their human capital (Hanushek and Woessmann 2008; Filmer et al. 2020). Furthermore, structural characteristics of the educational systems and macroeconomic crises generate regularly a reduction in instructional time and a great amount of variance in the quality of educational institutions (Abadzi 2009). To corroborate these findings, measuring the learning gap caused by COVID-19 with standardized test scores or similar qualitative measures as soon as the data is available, remains a very important issue.

### 5 Conclusions

School closures and other lockdown policies seem to have been able to reduce the mortality associated to COVID-19 in most countries (e.g. Dehning et al. 2020; Flaxman et al. 2020). On the other hand, they caused disruptions with potentially significant and serious long-run

[22] Interestingly, in Guatemala and Honduras, where high-school completion rates are very low among children with low-educated parents, the first robustness check (setting out the effect for those with less than completed secondary education) yields lower slope coefficients in the post-pandemic counterfactual with respect to the baseline.

consequences. In this paper, we quantify one dimension of these disruptions. Namely, the effect of the pandemic on inequality in educational attainment, and intergenerational persistence. Our projections show that the COVID-19 pandemic puts at risk the educational attainments of disadvantaged individuals and may cause a substantial decrease in intergenerational mobility. For instance, our estimates show that the average slope coefficient of intergenerational education persistence could rise by 7% from a regional average of 0.36 to 0.39. This number is significant, especially since in Latin America on average from 1940 to 1990 the slope coefficient has been decreasing by 4% from one four-year birth cohort to the next, respectively. Furthermore, high school completion rates of children with low-educated parents in Latin America could fall by 20 percentage points reversing decades of progress made by the region in terms of educational upward mobility.

Of course, our estimates do not take into account that future interventions may compensate for the learning losses in the near future. The desire for this to happen is, indeed, the main motivation for our analysis. We believe that strong measures to compensate education losses and risks for vulnerable children should be the priority at this point. Our projections show that without targeted policy measures, financial efforts, and political will to support education, the future of several generations of young Latin Americans is at serious danger. To avoid the irreversible destruction of the human capital of poor children and youth is a necessity and will define the shape of the society we will live in tomorrow.

**Supplementary Information** The online version contains supplementary material available at https://doi.org/10.1007/s10888-021-09501-x.

**Acknowledgments** This paper benefited greatly from the thoughtful suggestions of the editor, Philippe Van Kerm, and two anonymous referees. We are also grateful to Stephen Kaplan, Michael Wolfson, Raquel Fernandez, James Foster, Emilia Del Bono, and Daniel Heymann, as well as the participants of presentations at the IMF, ZEW, Universidad de San Andres, Universidad Nacional de Cordoba, ISER, and the UNDP-CEQ Institute-CGEP-SEGIB group of experts "The economy of the pandemic and social protection in Latin America". We would like to thank Melanie Gross, Valentina Martinez Pabón, and Facundo Pernigotti for their excellent research assistance. Guido Neidhöfer acknowledges funding by the Volkswagen Foundation (Project "Analyses of the Costs and Benefits of School Closures for the Containment of COVID-19").

**Funding** Open Access funding enabled and organized by Projekt DEAL.

**Open Access** This article is licensed under a Creative Commons Attribution 4.0 International License, which permits use, sharing, adaptation, distribution and reproduction in any medium or format, as long as you give appropriate credit to the original author(s) and the source, provide a link to the Creative Commons licence, and indicate if changes were made. The images or other third party material in this article are included in the article's Creative Commons licence, unless indicated otherwise in a credit line to the material. If material is not included in the article's Creative Commons licence and your intended use is not permitted by statutory regulation or exceeds the permitted use, you will need to obtain permission directly from the copyright holder. To view a copy of this licence, visit http://creativecommons.org/licenses/by/4.0/.

## Supplementary Information

ESM 1 (DOCX 1371 kb)
