# Supplementary material for: Intergenerational transmission of lockdown consequences: prognosis of the longer-run persistence of COVID-19 in Latin America
Source: J Econ Inequal. 2021 Jul 31;19(3):571–98. doi: 10.1007/s10888-021-09501-x (PMC8325400; doi:10.1007/s10888-021-09501-x)
Supplement: Supplementary file 1 — (DOCX 1371 kb) [file 10888_2021_9501_MOESM1_ESM.docx]

**Intergenerational transmission of lockdown consequences:**

**Prognosis of the longer-run persistence of COVID-19 in Latin America**

Guido Neidhöfer, Nora Lustig, Mariano Tommasi

**Supplemental Material**

**for online publication only**

Table S 1 - Intergenerational persistence measured by the slope coefficient before and after imputation of the COVID-19 shock on education. Cohort 1987-1994

|  |  | Counterfactual exercise | |  | Worst case scenario | |
| --- | --- | --- | --- | --- | --- | --- |
|  | $\beta$ | $\beta$ | in % |  | $\beta$ | in % |
| Argentina | 0.36 | 0.39 | -6.66 |  | 0.43 | -18.44 |
| Bolivia | 0.40 | 0.44 | -8.89 |  | 0.46 | -15.30 |
| Brazil | 0.27 | 0.30 | -11.23 |  | 0.34 | -23.15 |
| Chile | 0.35 | 0.37 | -6.58 |  | 0.41 | -17.90 |
| Colombia | 0.49 | 0.52 | -5.83 |  | 0.56 | -13.41 |
| Costa Rica | 0.32 | 0.34 | -8.01 |  | 0.38 | -18.61 |
| Dominican Rep. | 0.27 | 0.29 | -5.05 |  | 0.33 | -19.33 |
| Ecuador | 0.32 | 0.34 | -5.82 |  | 0.38 | -20.43 |
| El Salvador | 0.41 | 0.44 | -6.53 |  | 0.46 | -11.48 |
| Guatemala | 0.48 | 0.50 | -3.33 |  | 0.52 | -7.37 |
| Honduras | 0.56 | 0.57 | -1.76 |  | 0.59 | -5.06 |
| Mexico | 0.30 | 0.34 | -12.27 |  | 0.36 | -20.27 |
| Panama | 0.33 | 0.36 | -10.05 |  | 0.38 | -16.44 |
| Paraguay | 0.39 | 0.43 | -8.40 |  | 0.46 | -16.77 |
| Peru | 0.39 | 0.43 | -9.70 |  | 0.45 | -16.07 |
| Uruguay | 0.36 | 0.38 | -5.46 |  | 0.42 | -18.73 |
| Venezuela | 0.18 | 0.22 | -19.00 |  | 0.25 | -36.67 |

Notes: Worst case scenario shows an instructional loss equivalent to 100% of the school year without any compensatory effect of mitigation policies. Source: Latinobarometro, own estimates.

Table S 2 - Microsimulation of household income losses by level of education of the household head.

| Households losing >50% of their income (in %) | |  |  |  |  |
| --- | --- | --- | --- | --- | --- |
| **Without mitigation programs** | |  |  |  |  |
|  | Level of education |  | | | |
|  |  | Argentina | Brazil | Colombia | Mexico |
|  | Illiterate | 6.1 | 5.5 | 9.6 | 6.4 |
|  | Incomplete primary | 9.2 | 8.9 | 10.1 | 8.5 |
|  | Complete primary | 14.2 | 10.3 | 14.1 | 11.3 |
|  | Incomplete Secondary | 15.8 | 12.3 | 17.0 | 13.2 |
|  | Complete Secondary | 13.3 | 10.0 | 15.3 | 10.0 |
|  | Incomplete Tertiary | 10.0 | 8.7 | 12.3 | 7.3 |
|  | Complete Tertiary | 7.8 | 4.1 | 9.5 | 5.5 |
| **With mitigation programs** | |  |  |  |  |
|  | Level of education |  | | | |
|  |  | Argentina | Brazil | Colombia | Mexico |
|  | Illiterate | 5.6 | 4.5 | 9.4 | 6.4 |
|  | Incomplete primary | 8.4 | 7.4 | 9.8 | 8.5 |
|  | Complete primary | 12.1 | 8.8 | 13.8 | 11.3 |
|  | Incomplete Secondary | 13.4 | 10.5 | 16.7 | 13.2 |
|  | Complete Secondary | 12.3 | 9.0 | 15.1 | 10.0 |
|  | Incomplete Tertiary | 9.3 | 8.2 | 12.3 | 7.3 |
|  | Complete Tertiary | 7.4 | 3.7 | 9.5 | 5.5 |

Notes: Values obtained by microsimulation. For more details on the method and data sources see Lustig et al. (2020).

Figure S 1 - Intergenerational persistence of education in Latin America before and after imputation of the COVID-19 shock on human capital.


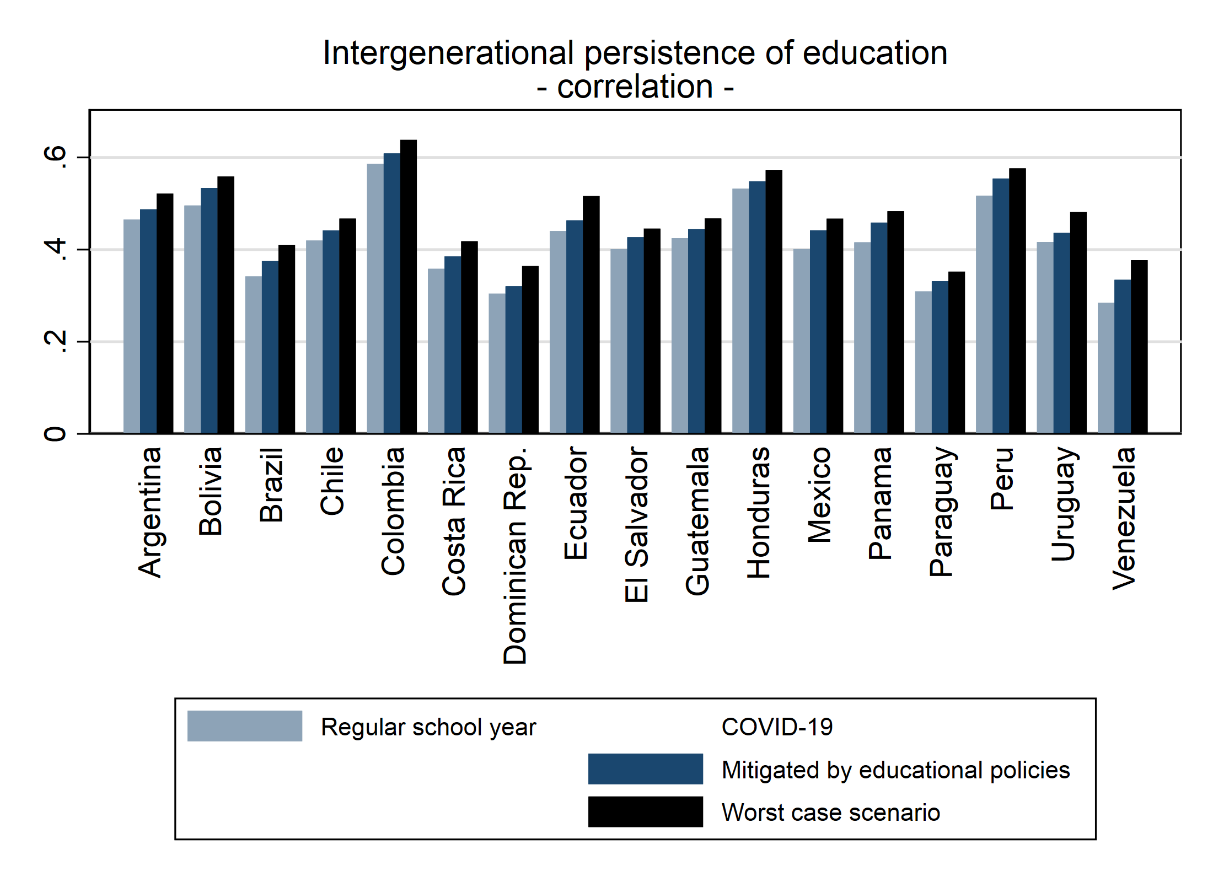

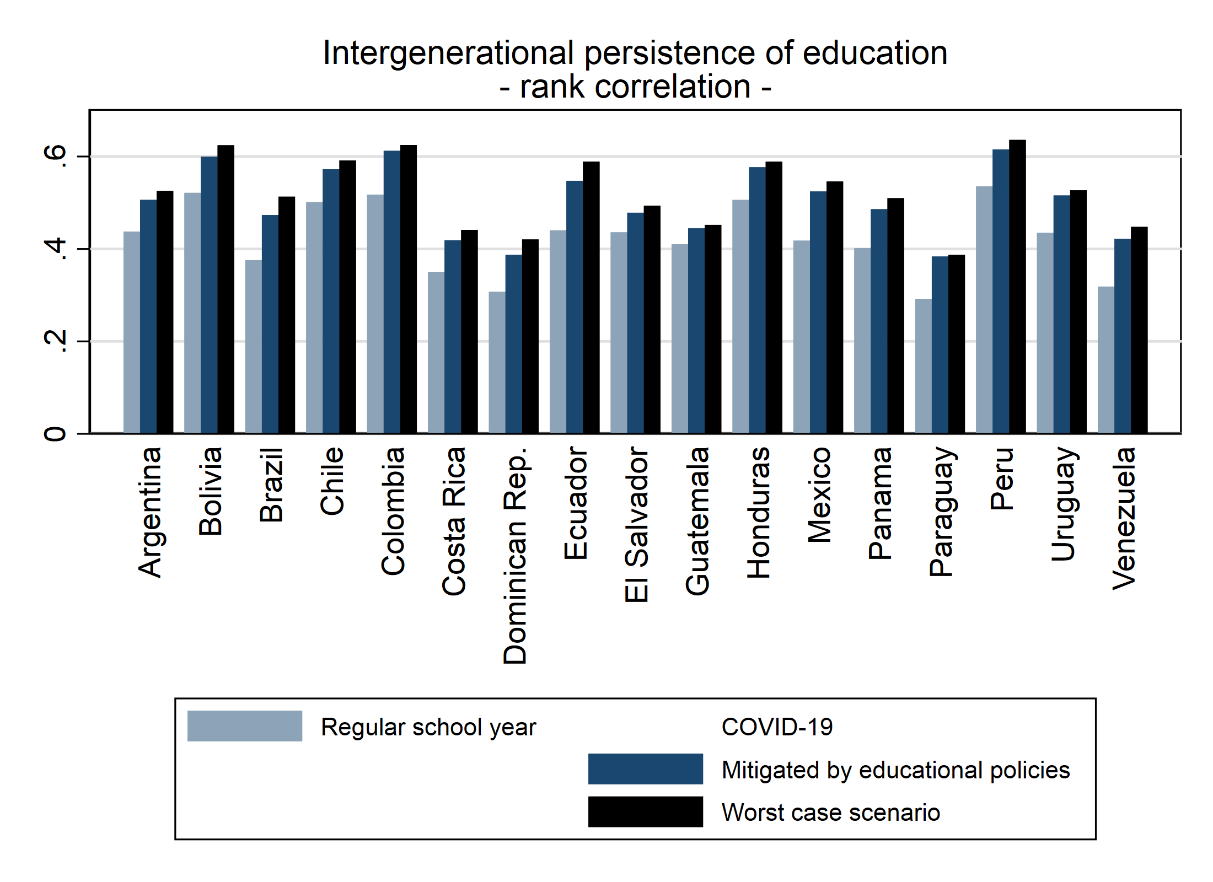


Notes: Worst case scenario shows an instructional loss equivalent to 100% of the school year without any compensatory effect of mitigation policies. Source: Latinobarometro, own estimates.

Figure S 2 - Estimated likelihood to complete secondary education by socioeconomic background before and after imputation of the COVID-19 shock. Scenario of dispersed educational losses.


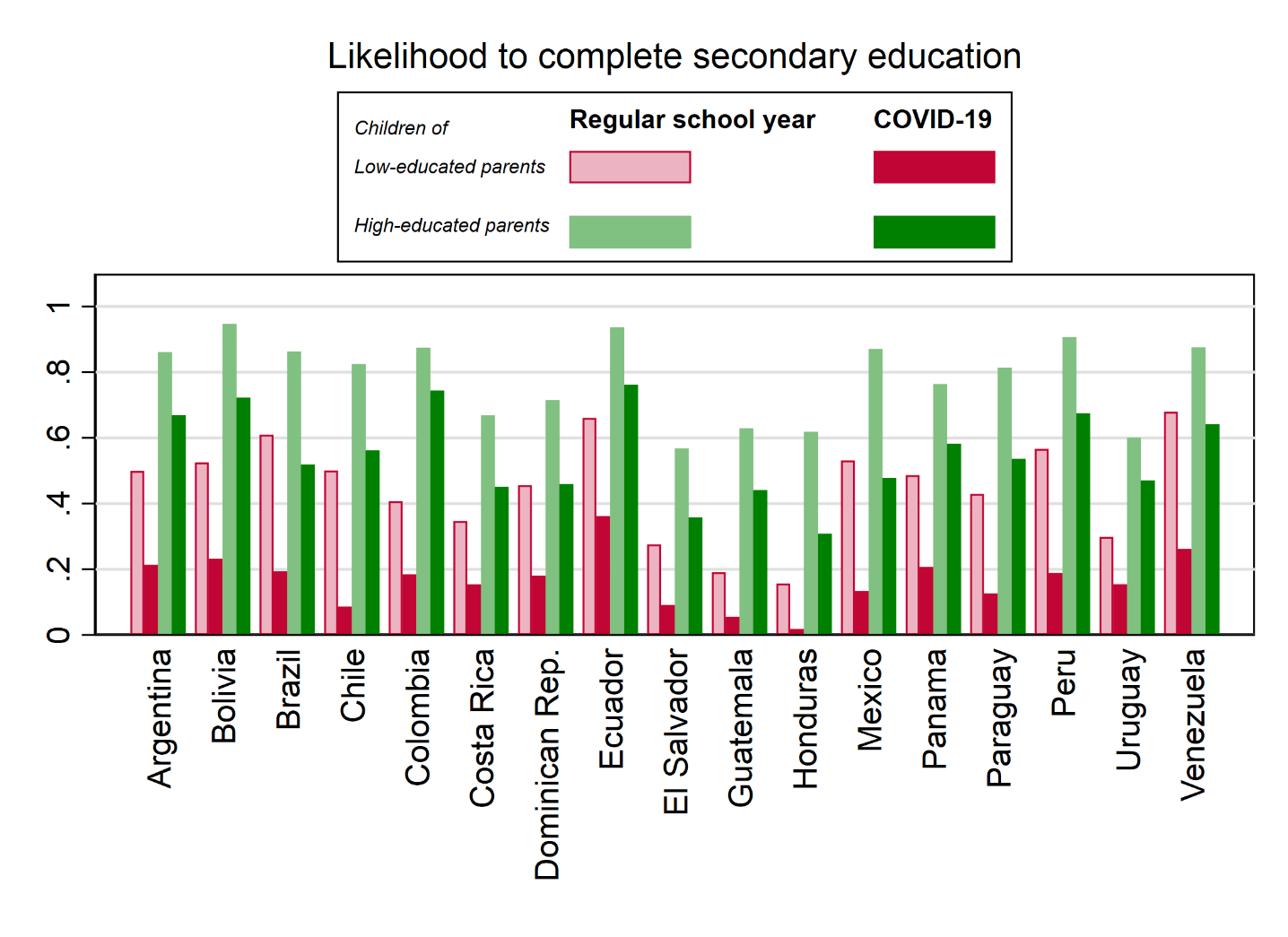


Notes: Bars show the likelihood to complete at least 12 years of schooling before and after simulation of the COVID-19 shock on education. High educated parents have at least completed secondary education, low educated parents less than completed secondary education. Source: Latinobarometro, own estimates.

Figure S 3 - Estimated likelihood to complete secondary education by socioeconomic background before and after imputation of the COVID-19 shock. Robustness.


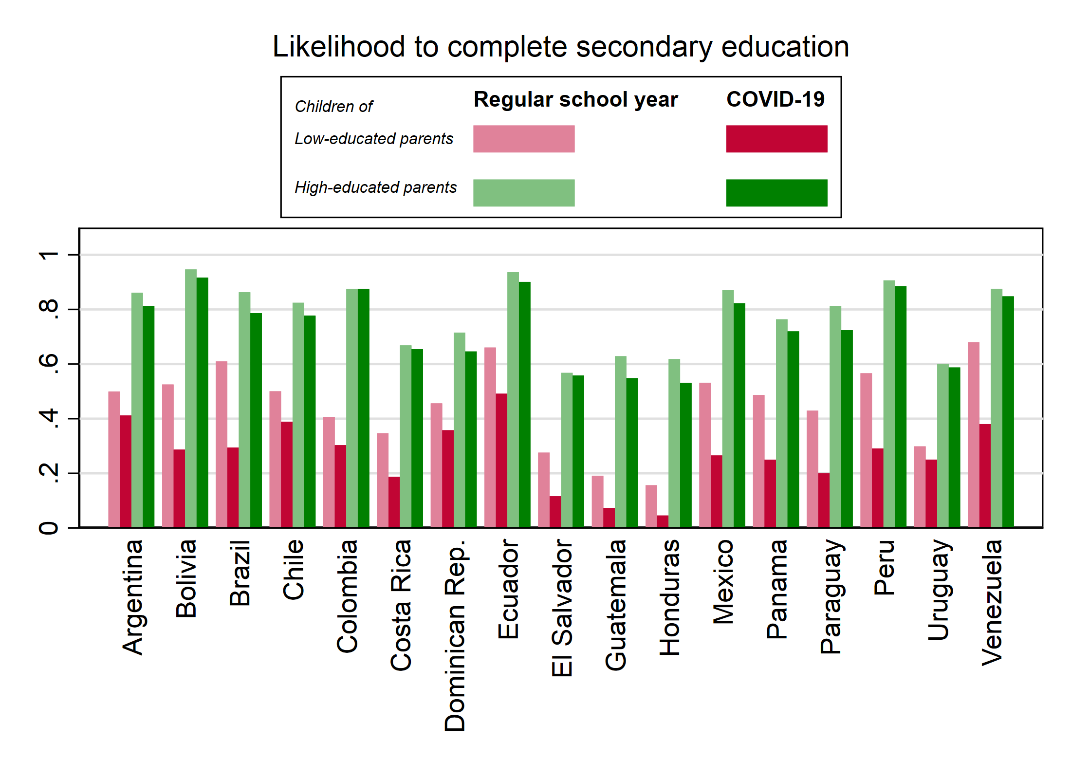


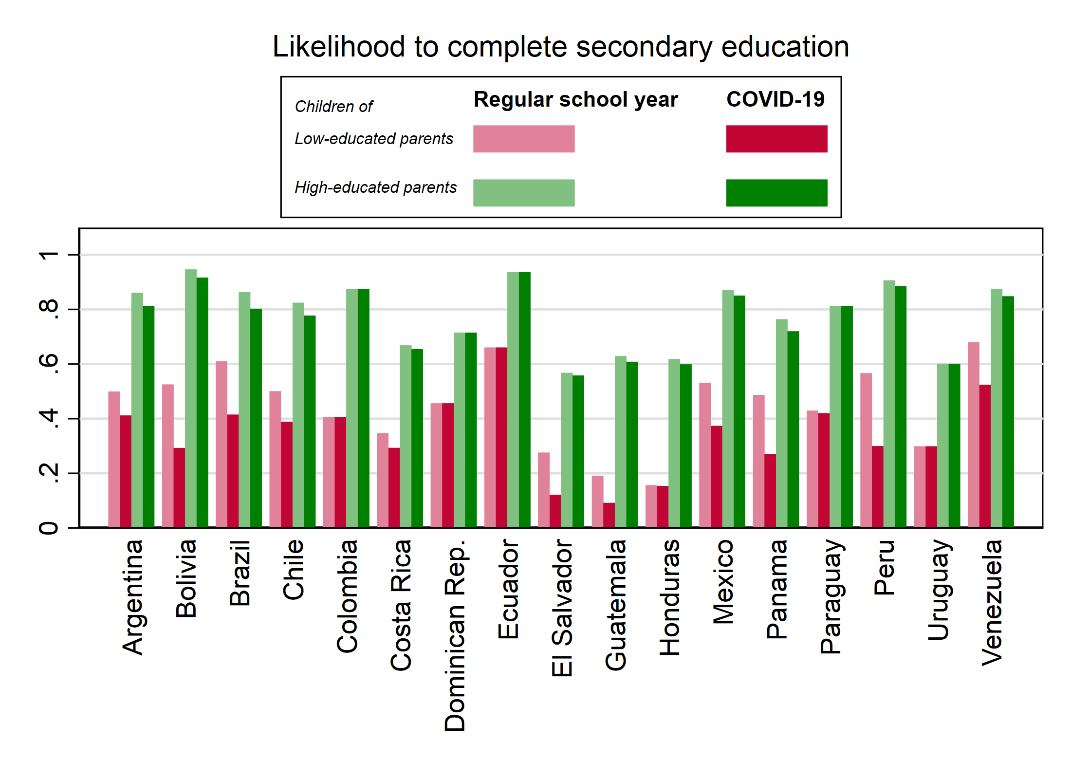


Notes: Bars show the likelihood to complete at least 11.75 years of schooling (upper graph) or 11.5 years of schooling (lower graph) before and after simulation of the COVID-19 shock on education. High educated parents have at least completed secondary education, low educated parents less than completed secondary education. Source: Latinobarometro, own estimates.

Table S 3 – Indicators used to estimate instructional loss

| **Indicator** | **Sources** | **Brief explanation** |
| --- | --- | --- |
| Days of school closure | Argentina: ^[[1]](#endnote-1)^, ^[[2]](#endnote-2)^  Bolivia: ^[[3]](#endnote-3)^, ^[[4]](#endnote-4)^  Brasil: ^[[5]](#endnote-5)^, ^[[6]](#endnote-6)^  Chile: ^[[7]](#endnote-7)^, ^[[8]](#endnote-8)^  Colombia: ^[[9]](#endnote-9)^, ^[[10]](#endnote-10)^  Costa Rica: ^[[11]](#endnote-11)^, ^[[12]](#endnote-12)^  Dominican Republic: ^[[13]](#endnote-13)^, ^[[14]](#endnote-14)^, ^[[15]](#endnote-15)^  Ecuador: ^[[16]](#endnote-16)^, ^[[17]](#endnote-17)^, ^[[18]](#endnote-18)^  El Salvador: ^[[19]](#endnote-19)^. ^[[20]](#endnote-20)^  Guatemala: ^[[21]](#endnote-21)^, ^[[22]](#endnote-22)^  Honduras: ^[[23]](#endnote-23)^, ^[[24]](#endnote-24)^  Mexico: ^[[25]](#endnote-25)^, ^[[26]](#endnote-26)^  Nicaragua: ^[[27]](#endnote-27)^  Panama: ^[[28]](#endnote-28)^, ^[[29]](#endnote-29)^  Paraguay: ^[[30]](#endnote-30)^, ^[[31]](#endnote-31)^  Peru: ^[[32]](#endnote-32)^, ^[[33]](#endnote-33)^  Uruguay: ^[[34]](#endnote-34)^, ^[[35]](#endnote-35)^  Venezuela: ^[[36]](#endnote-36)^, ^[[37]](#endnote-37)^ | Number of lost school days since the date of school closure (taking into account school reopenings). Weekends and holidays were not counted as lost school days. |
| Total days of school in normal school year | Argentina: ^[[38]](#endnote-38)^  Bolivia: ^[[39]](#endnote-39)^  Brasil:^[[40]](#endnote-40)^  Chile: ^[[41]](#endnote-41)^  Colombia: ^[[42]](#endnote-42)^  Costa Rica: ^[[43]](#endnote-43)^  Dominican Republic: ^[[44]](#endnote-44)^  Ecuador: ^[[45]](#endnote-45)^  El Salvador: ^[[46]](#endnote-46)^  Guatemala: ^[[47]](#endnote-47)^  Honduras: ^[[48]](#endnote-48)^  Mexico: ^[[49]](#endnote-49)^  Nicaragua: ^[[50]](#endnote-50)^  Panama: ^[[51]](#endnote-51)^  Paraguay: ^[[52]](#endnote-52)^  Peru: ^[[53]](#endnote-53)^  Uruguay: ^[[54]](#endnote-54)^  Venezuela: ^[[55]](#endnote-55)^ | Number of mandatory school days during a regular academic year. |
| Internet coverage by socioeconomic group | World Bank open data *Individuals using the Internet (% of population)*  Household surveys^[[56]](#endnote-56)^:  Argentina:^[[57]](#endnote-57)^  Bolivia:^[[58]](#endnote-58)^  Chile:^[[59]](#endnote-59)^  Colombia:^[[60]](#endnote-60)^  Costa Rica:^[[61]](#endnote-61)^  Ecuador:^[[62]](#endnote-62)^  El Salvador:^[[63]](#endnote-63)^  Mexico:^[[64]](#endnote-64)^  Paraguay:^[[65]](#endnote-65)^  Peru:^[[66]](#endnote-66)^  Uruguay:^[[67]](#endnote-67)^ | For the percentage of internet access by country we used a harmonized and standardized dataset provided by the World Bank. To estimate the distribution across groups, we used the last household survey available for each country.  We estimated internet access within each socioeconomic group (by education of the head of household) as the percentage of families with internet access, among those with children and whose head of household had the corresponding educational level. Internet access by group was then re-scaled so that when all groups were aggregated the percentage would match the World Bank estimates. |
| Offline learning / Home schooling | Argentina: ^[[68]](#endnote-68)^, ^[[69]](#endnote-69)^  Bolivia: ^[[70]](#endnote-70)^,^[[71]](#endnote-71)^  Brasil:^[[72]](#endnote-72)^,^[[73]](#endnote-73)^,^[[74]](#endnote-74)^  Chile: ^[[75]](#endnote-75)^, ^[[76]](#endnote-76)^  Colombia: ^[[77]](#endnote-77)^  Costa Rica:^[[78]](#endnote-78)^, ^[[79]](#endnote-79)^  Dominican Republic: ^[[80]](#endnote-80)^  Ecuador: ^[[81]](#endnote-81)^  El Salvador: ^[[82]](#endnote-82)^, ^[[83]](#endnote-83)^  Guatemala: ^[[84]](#endnote-84)^  Honduras: ^[[85]](#endnote-85)^  Mexico: ^[[86]](#endnote-86)^  Nicaragua:  Panama: ^[[87]](#endnote-87)^, ^[[88]](#endnote-88)^  Paraguay: ^[[89]](#endnote-89)^  Peru: ^[[90]](#endnote-90)^  Uruguay: ^[[91]](#endnote-91)^, ^[[92]](#endnote-92)^  Venezuela: ^[[93]](#endnote-93)^ | Governments made different efforts for children to keep on studying during the pandemic. Mainly, they gave out printed copies, sent educational material via cellphone, and broadcasted educational contents through radio and TV. We built dummy variables for cellphone education, printed copies, TV and radio programs, and then calculated an index for each country. The index $f$ was calculated as a simple average between the dummies. The index is one if all these educational tools were used by the country’s education system during the school closure, and zero if none of them was used. For all other combinations the index lies between these values. |
| Online learning | CIMA & IADB  Nota 20, *COVID-19: ¿Estamos preparados para el aprendizaje en línea?* | On top of the efforts previously mentioned, governments also provided resources or policies for online learning. Using the data from CIMA and IDB, we constructed an index taking into account Digital Platforms, Virtual Tutoring, Digital Resources And Digital Content Repositories. The index $n$ captures the use of these tools by the country’s educational system and was calculated as a simple average. The index is one if all these educational tools were used by the country’s education system during the school closure, and zero if none of them was used. For all other combinations the index lies between these values. |
| COVID-19 cases and deaths per inhabitant | European Centre for Disease Prevention and Control | The number of cases and deaths reported by countries is divided by the population size in 2019. |
| Average household size | United Nations, Department  of Economic and Social Affairs, Population Division (2017).  Household Size and Composition  Around the World 2017 – Data Booklet  (ST/ESA/  SER.A/405). | Average number of people living in the same household. |

Figure S 4 – Educational upward mobility and top persistence of female and male


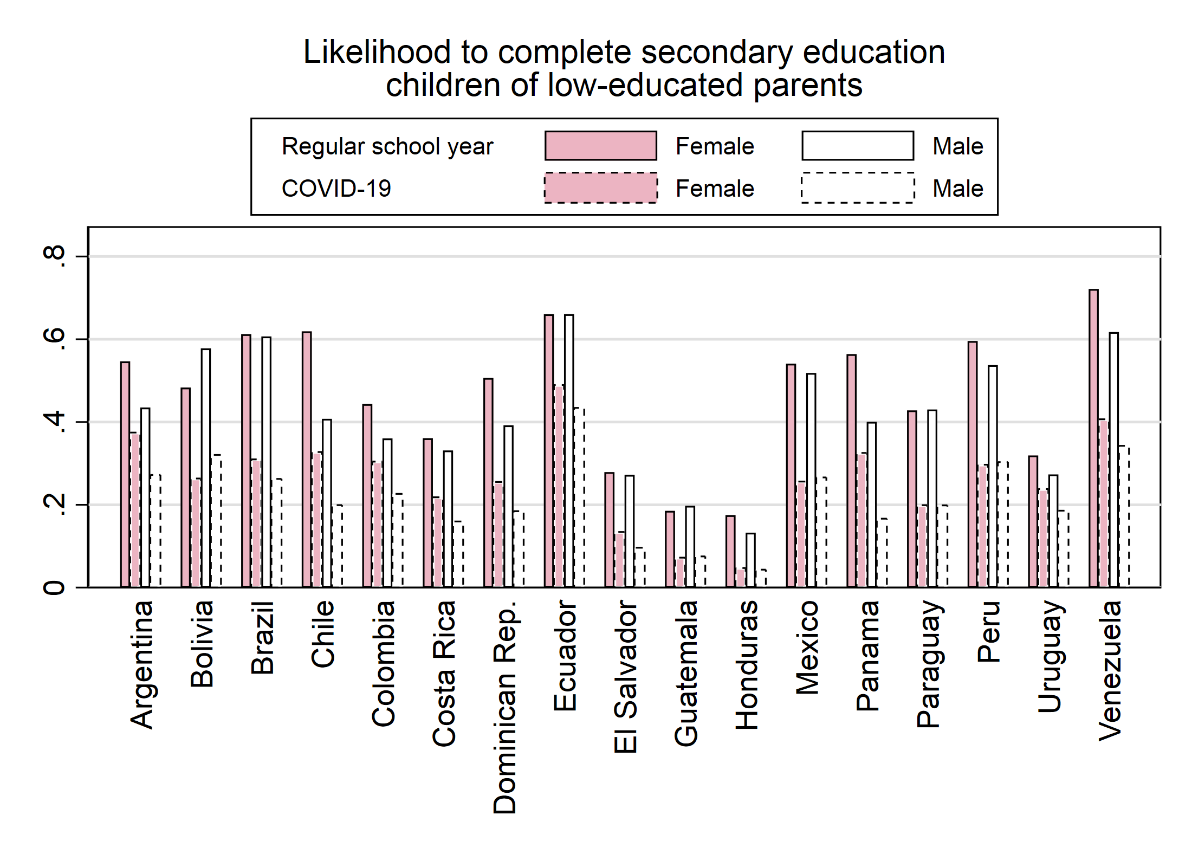


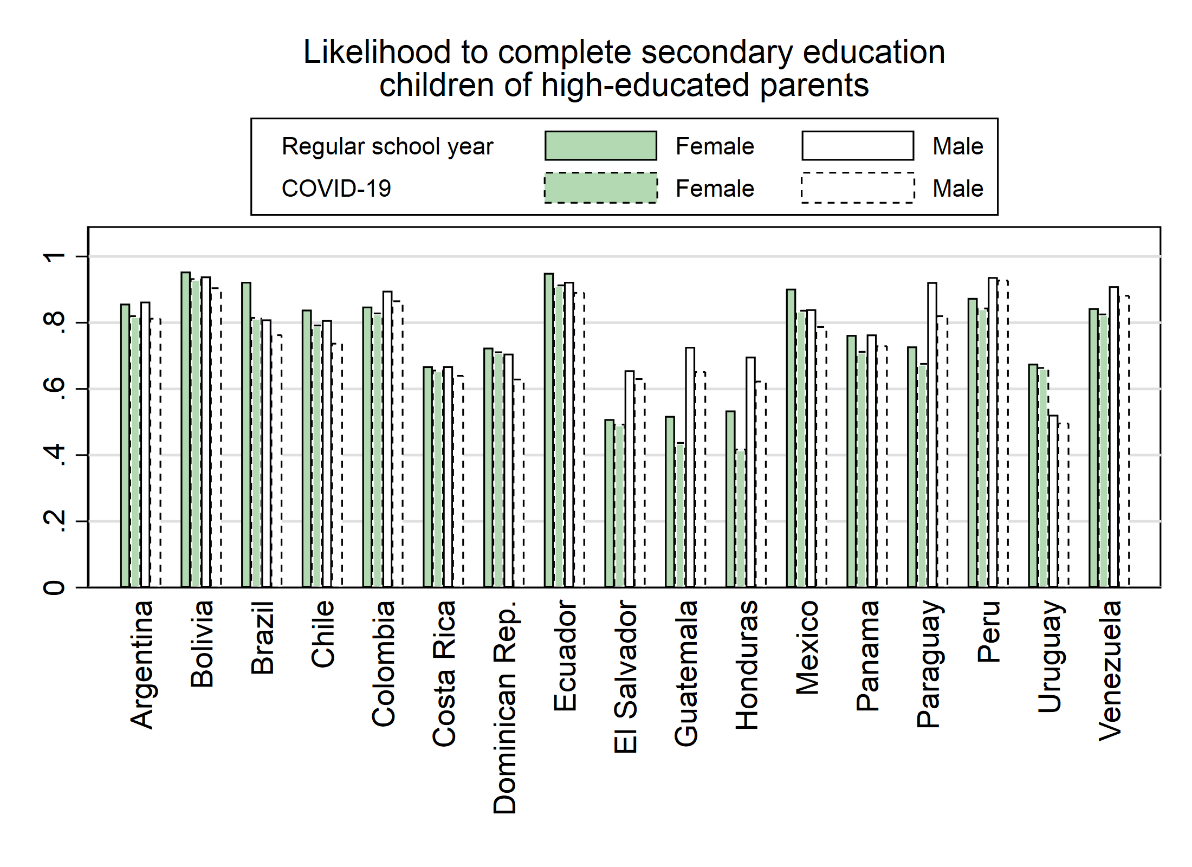


Notes: Bars show the likelihood to complete at least 12 years of schooling before and after simulation of the COVID-19 shock on education. High educated parents have at least completed secondary education, low educated parents less than completed secondary education. Source: Latinobarometro, own estimates.

Figure S 5 – Point estimates for baseline and alternative specifications

(a) Slope coefficient


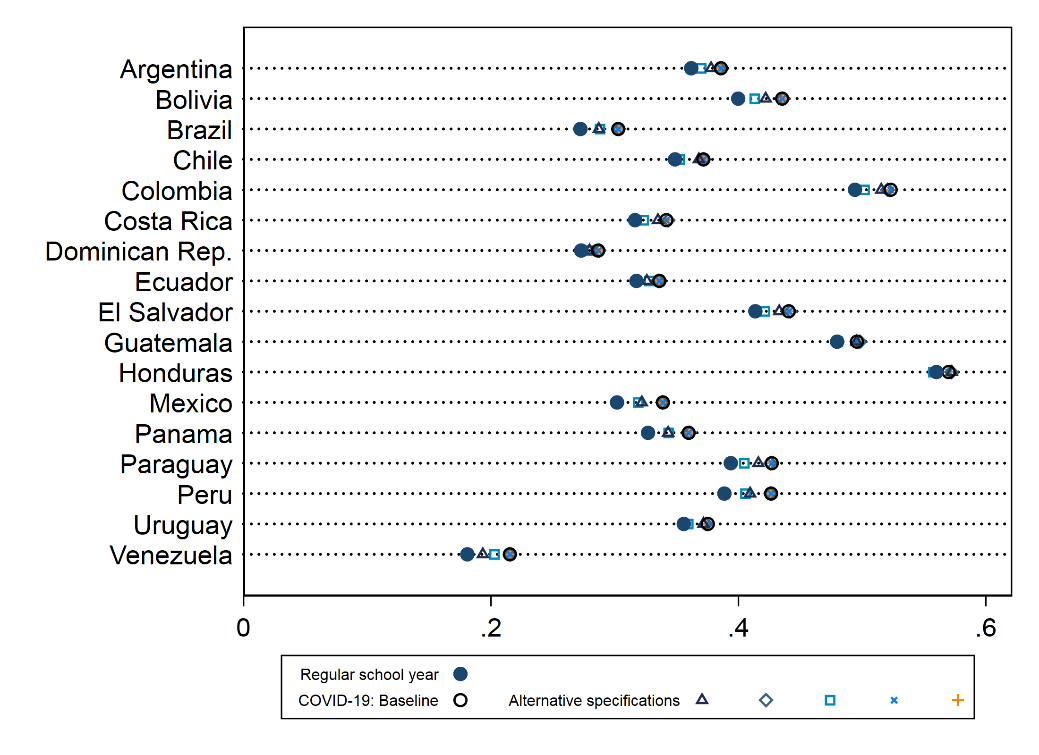


(b) Absolute upward mobility


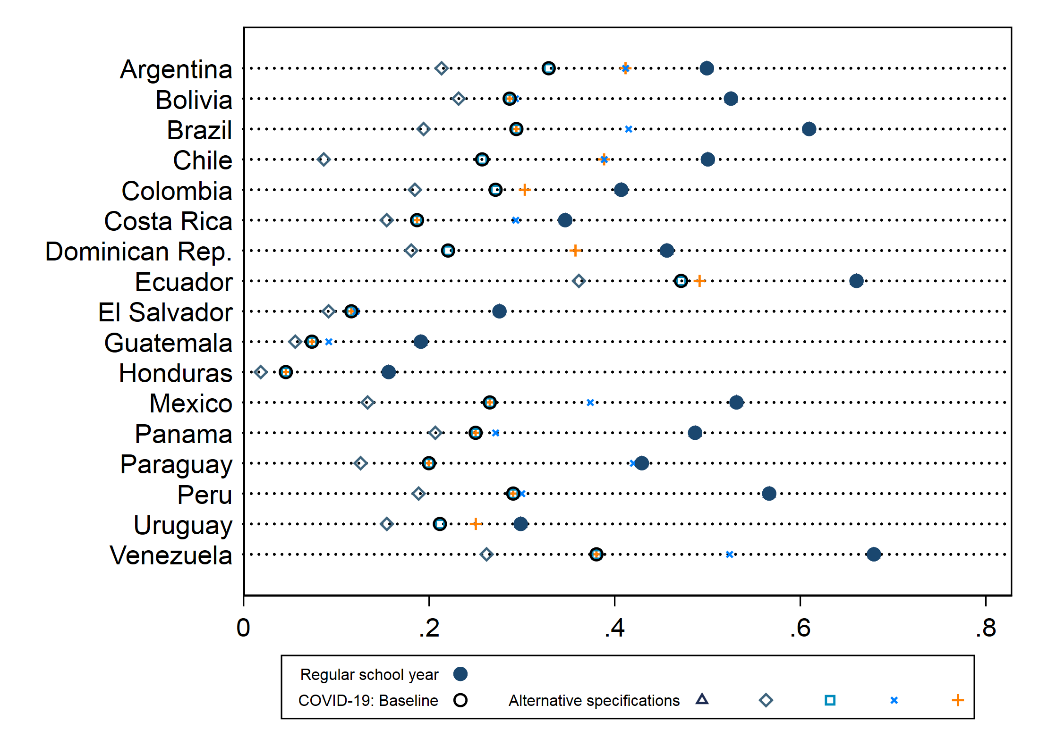


Notes: Dots show the point estimates of the mobility indexes for the regular school year and for each specification of the COVID-19 counterfactual. Source: Latinobarometro, own estimates.

Table S 4 – Distribution of parental education in each country – weighted shares in %

|  | Parental education | | | | | | |
| --- | --- | --- | --- | --- | --- | --- | --- |
| Country | Illiterate | Incomplete primary | Complete primary | Incomplete secondary | Complete secondary | Incomplete tertiary | Complete tertiary |
| Argentina | 2.63 | 8.82 | 32.96 | 10.26 | 26.42 | 3.03 | 15.88 |
| Bolivia | 20.74 | 35.28 | 5.27 | 9.43 | 16.86 | 2.53 | 9.89 |
| Brazil | 8.37 | 39.13 | 12.89 | 3.62 | 24.85 | 1.63 | 9.51 |
| Chile | 0.64 | 20.92 | 5.55 | 17.87 | 38.68 | 2.61 | 13.73 |
| Colombia | 8.51 | 26.4 | 11.41 | 14.56 | 19.67 | 1.07 | 18.38 |
| Costa Rica | 19.96 | 4.05 | 34.42 | 11.42 | 19.66 | 4.47 | 6.03 |
| Dominican Rep. | 21.42 | 18.04 | 15.32 | 11.41 | 22.6 | 3.07 | 8.14 |
| Ecuador | 6.68 | 7.32 | 36.02 | 8.27 | 25.3 | 5.33 | 11.08 |
| El Salvador | 33.07 | 30.14 | 14.59 | 1.91 | 14.34 | 0.95 | 4.99 |
| Guatemala | 49.69 | 14.92 | 20.09 | 6.12 | 4.18 | 1.51 | 3.48 |
| Honduras | 36.36 | 21.14 | 26.19 | 3.39 | 10.02 | 0.43 | 2.48 |
| Mexico | 16.14 | 12.8 | 23.31 | 9.57 | 23.89 | 3.71 | 10.58 |
| Nicaragua | 39.48 | 16.46 | 15.25 | 11 | 11.8 | 1.4 | 4.61 |
| Panama | 23.75 | 2.98 | 22.49 | 10.38 | 25.25 | 1.94 | 13.21 |
| Paraguay | 5.43 | 74.57 | 2.53 | 2.63 | 11.69 | 1.53 | 1.62 |
| Peru | 12.38 | 11.75 | 13.25 | 6.4 | 33.08 | 3.86 | 19.3 |
| Uruguay | 1.01 | 5.23 | 33.75 | 28.19 | 15.69 | 5.88 | 10.25 |
| Venezuela | 8.23 | 4.38 | 27.21 | 11.19 | 32.76 | 2.39 | 13.85 |
| Latin America | 17.31 | 20.26 | 19.4 | 9.57 | 20.93 | 2.64 | 9.89 |

Notes: Values in each cell show the weighted share in percentage of individuals for each category of parental education. Parental education is the education of the parent with the highest level of education among the two. Sample includes individuals born between 1987 and 1994. Source: Latinobarometro, own estimates.

Table S 5 – Transition Matrix

| Child’s education | Parental education | | | | | | |
| --- | --- | --- | --- | --- | --- | --- | --- |
|  | Illiterate | Incomplete primary | Complete primary | Incomplete secondary | Complete secondary | Incomplete tertiary | Complete tertiary |
| Illiterate | 21.81 | 4.41 | 2.69 | 1.18 | 0.5 | 0.24 | 0.84 |
| Incomplete primary | 20.51 | 22.2 | 6.8 | 5.55 | 3.47 | 1.67 | 0.72 |
| Complete primary | 19.98 | 18.97 | 19.69 | 9.01 | 7.11 | 3.57 | 2.76 |
| Incomplete Secondary | 11.49 | 14.97 | 21.33 | 24.07 | 10.45 | 12.33 | 7.79 |
| Complete Secondary | 17.31 | 27.15 | 29.24 | 35.63 | 34.22 | 21.48 | 19.75 |
| Incomplete tertiary | 4.64 | 8.02 | 12.33 | 15.37 | 23.83 | 39.84 | 29.35 |
| Complete tertiary | 4.25 | 4.29 | 7.91 | 9.19 | 20.43 | 20.88 | 38.8 |

Notes: Values in each cell show the weighted share in percentage of individuals for each category of parental education. Parental education is the education of the parent with the highest level of education among the two. Sample includes individuals born between 1987 and 1994. Source: Latinobarometro, own estimates.

**Table S 6 – Distribution of education by parental background**

| Children of… | low-educated parents | | | high-educated parents | | |
| --- | --- | --- | --- | --- | --- | --- |
| Share with… | less than completed secondary | completed secondary | at least some tertiary | less than completed secondary | completed secondary | at least some tertiary |
| Argentina | 50.07 | 28.59 | 21.34 | 13.94 | 26.1 | 59.96 |
| Bolivia | 47.47 | 29.36 | 23.17 | 5.37 | 30.12 | 64.51 |
| Brazil | 39.05 | 41.55 | 19.4 | 13.69 | 42.52 | 43.8 |
| Chile | 49.96 | 41.39 | 8.64 | 17.56 | 29.58 | 52.86 |
| Colombia | 59.3 | 22.24 | 18.46 | 12.57 | 16.49 | 70.95 |
| Costa Rica | 65.34 | 19.24 | 15.43 | 33.15 | 27.16 | 39.69 |
| Dominican Rep. | 54.37 | 27.56 | 18.07 | 28.53 | 31.59 | 39.87 |
| Ecuador | 33.94 | 29.92 | 36.14 | 6.38 | 22.35 | 71.27 |
| El Salvador | 72.42 | 18.42 | 9.16 | 43.22 | 25.69 | 31.09 |
| Guatemala | 80.89 | 13.54 | 5.57 | 37.12 | 26.32 | 36.56 |
| Honduras | 84.36 | 13.79 | 1.85 | 38.18 | 38.95 | 22.87 |
| Mexico | 46.88 | 39.75 | 13.37 | 12.95 | 47.04 | 40.01 |
| Nicaragua | 75.29 | 17.3 | 7.41 | 50.29 | 22.41 | 27.31 |
| Panama | 51.34 | 28 | 20.67 | 23.63 | 26.35 | 50.03 |
| Paraguay | 57.08 | 30.31 | 12.62 | 18.67 | 29.92 | 51.41 |
| Peru | 43.35 | 37.78 | 18.87 | 9.37 | 27.69 | 62.95 |
| Uruguay | 70.13 | 14.43 | 15.44 | 39.94 | 18.17 | 41.9 |
| Venezuela | 32.07 | 41.74 | 26.19 | 12.49 | 30.24 | 57.27 |
| Total | 58.08 | 26.42 | 15.5 | 18.45 | 28.94 | 52.61 |

Notes: Values in each cell show the weighted share. Parental education is the education of the parent with the highest level of education among the two. High-educated parents have at least a completed secondary degree, low-educated parents less than a completed secondary degree. Sample includes individuals born between 1987 and 1994. Source: Latinobarometro, own estimates.

Figure S 6 - Estimated likelihood to complete secondary education by socioeconomic background before and after imputation of the COVID-19 shock. Different threshold for parental education.


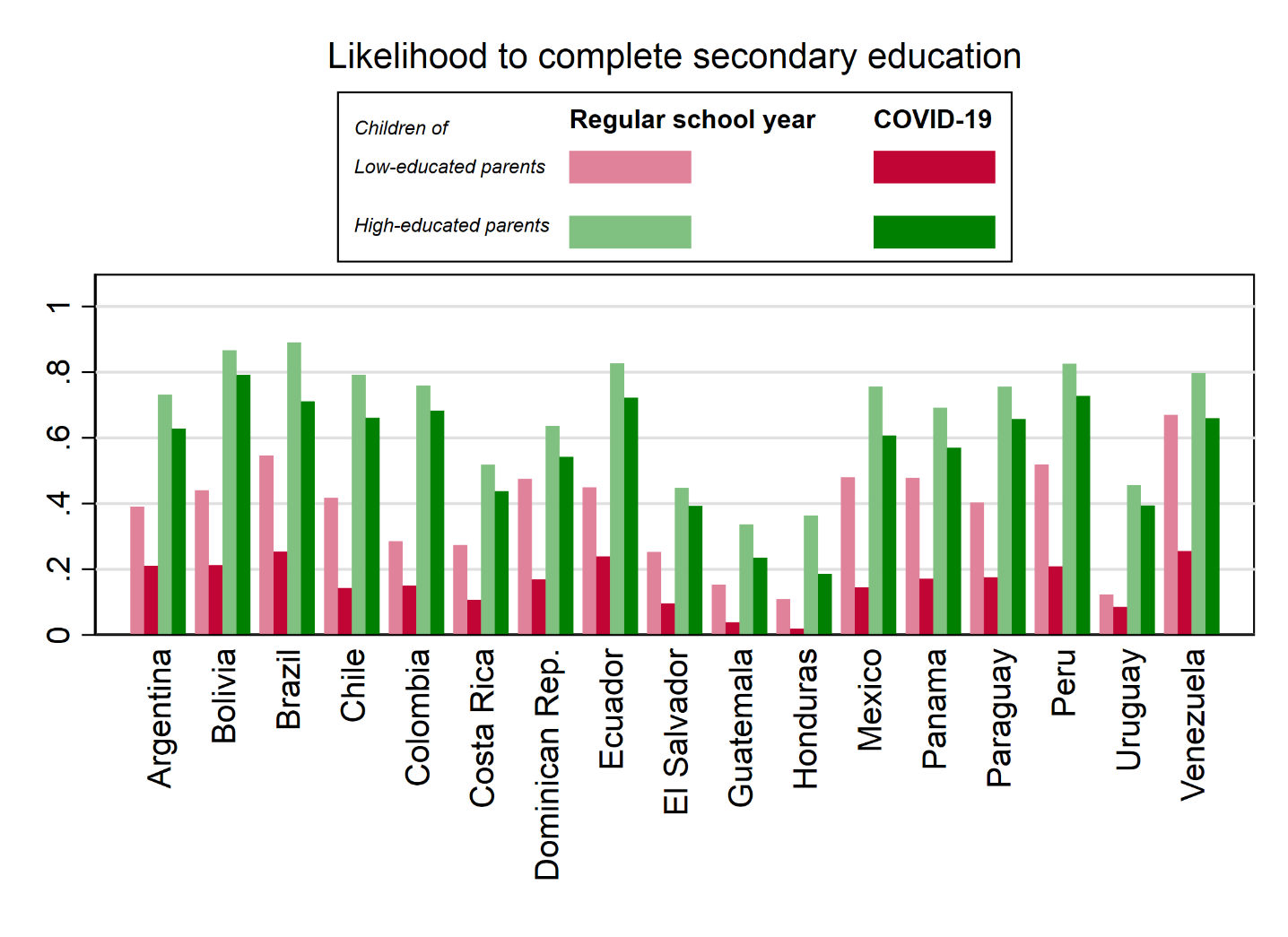


Notes: Bars show the likelihood to complete at least 12 years of schooling before and after simulation of the COVID-19 shock on education. High educated parents have at least completed primary education, low educated parents less than completed primary education. Source: Latinobarometro, own estimates.

1. <https://www.argentina.gob.ar/noticias/coronavirus-suspension-de-clases-presenciales-14-dias-consecutivos-partir-del-16-de-marzo> [↑](#endnote-ref-1)
2. <https://www.argentina.gob.ar/sites/default/files/res_cfe_361_19.pdf> [↑](#endnote-ref-2)
3. <http://www.presidencia.gob.bo/index.php/prensa/noticias/1235-coronavirus-bolivia-suspende-labores-educativas-vuelos-desde-y-hacia-europa-e-intensifica-el-control-en-fronteras> [↑](#endnote-ref-3)
4. <https://www.minedu.gob.bo/files/documentos-normativos/resoluciones-ministeriales/2020/001-REGULAR.pdf> [↑](#endnote-ref-4)
5. <https://www.efe.com/efe/america/sociedad/brasil-se-prepara-para-el-inicio-de-las-restricciones-ante-avance-del-covid-19/20000013-4196413> [↑](#endnote-ref-5)
6. <http://www.dias-festivos.com/country/Brazil-Sao-Paulo_556.htm> [↑](#endnote-ref-6)
7. <https://www.mineduc.cl/coronavirus-mineduc-lanza-plataforma-aprendo-en-linea/> [↑](#endnote-ref-7)
8. <https://ayudamineduc.cl/ficha/calendarios-escolares-regionales> [↑](#endnote-ref-8)
9. <https://www.mineducacion.gov.co/portal/salaprensa/Noticias/393933:Gobierno-Nacional-anuncia-medidas-en-materia-educativa-para-ofrecer-garantias-de-salud-publica-a-la-comunidad> [↑](#endnote-ref-9)
10. <https://www.mineducacion.gov.co/portal/micrositios-preescolar-basica-y-media/Jornada-Escolar-2020/Calendario/392187:Calendario> [↑](#endnote-ref-10)
11. <https://www.ministeriodesalud.go.cr/index.php/centro-de-prensa/noticias/741-noticias-2020/1572-gobierno-declara-estado-de-emergencia-nacional-impide-llegada-de-extranjeros-y-se-suspende-lecciones-en-todos-los-centros-educativos-del-pais> [↑](#endnote-ref-11)
12. <https://publicholidays.co.cr/es/school-holidays/2020-dates/> [↑](#endnote-ref-12)
13. <https://www.expansion.com/empresas/transporte/2020/03/18/5e7185f7e5fdea9e608b4654.html> [↑](#endnote-ref-13)
14. <https://publicholidays.do/es/school-holidays/> [↑](#endnote-ref-14)
15. <https://eldominicano.do/ano-escolar-2020-2021-dividido-en-dos-horarios/> [↑](#endnote-ref-15)
16. <https://www.eluniverso.com/noticias/2020/03/12/nota/7778478/coronavirus-suspension-clases-ministerio-educacion> [↑](#endnote-ref-16)
17. <https://www.elcomercio.com/actualidad/inicio-clases-costa-covid19-ecuador.html> [↑](#endnote-ref-17)
18. <https://educacion.gob.ec/wp-content/uploads/downloads/2019/08/Cronograma-Sierra-Amazonia-2019-2020.pdf> [↑](#endnote-ref-18)
19. <https://www.mined.gob.sv/jdownloads/Circulares%20Institucionales/Circulares%202020/CIRCULAR%209-2020.pdf> [↑](#endnote-ref-19)
20. <https://www.mined.gob.sv/jdownloads/Calendario%20escolar/calendario2020.pdf> [↑](#endnote-ref-20)
21. <https://www.prensalibre.com/guatemala/comunitario/ciclo-escolar-seguira-suspendido-durante-todo-abril-por-el-coronavirus/> [↑](#endnote-ref-21)
22. <https://publicholidays.la/guatemala/es/school-holidays/2020-dates/> [↑](#endnote-ref-22)
23. <https://www.infobae.com/america/agencias/2020/03/12/honduras-suspende-clases-en-colegios-y-principal-universidad-cesa-operaciones-ante-coronavirus/> [↑](#endnote-ref-23)
24. <https://www.se.gob.hn/calendario-escolar/> [↑](#endnote-ref-24)
25. <https://elpais.com/sociedad/2020-03-14/mexico-adelanta-y-alarga-las-vacaciones-de-semana-santa-para-contener-la-epidemia.html> [↑](#endnote-ref-25)
26. <http://www.dias-festivos-mexico.com.mx/vacaciones-2020/> [↑](#endnote-ref-26)
27. <https://www.mined.gob.ni/estrategia-nacional-del-subsistema-de-educacion-basica-y-media-para-enfrentar-el-desafio-de-la-pandemia-covid-19/#dflip-df_15798/1/> [↑](#endnote-ref-27)
28. <https://www.oecd.org/coronavirus/country-policy-tracker/#Containmentmeasures> [↑](#endnote-ref-28)
29. <https://publicholidays.com.pa/es/school-holidays/2020-dates/> [↑](#endnote-ref-29)
30. <https://www.mec.gov.py/cms_v2/adjuntos/15716?1589908264> [↑](#endnote-ref-30)
31. <https://www.mec.gov.py/sigmec/resoluciones/6586-2019-PETTA.pdf> [↑](#endnote-ref-31)
32. <https://cholilaonline.com/2020/03/martin-vizcarra-anuncio-la-suspension-de-clases-en-peru-por-coronavirus.html> [↑](#endnote-ref-32)
33. <http://www.dias-festivos.com/country/Peru_135.htm> [↑](#endnote-ref-33)
34. <https://www.elpais.com.uy/informacion/politica/gobierno-suspende-clases-pais-semanas-coronavirus.html> [↑](#endnote-ref-34)
35. <https://www.presidencia.gub.uy/comunicacion/comunicacionnoticias/conferencia-lacalle-pou-retorno-clases> [↑](#endnote-ref-35)
36. <http://www.dias-festivos.com/country/Venezuela_186.htm> [↑](#endnote-ref-36)
37. Reglamento General de la Ley Orgánica de Educación. Decreto N° 313 Gaceta Oficial N° 36.787 (Reforma) del 16/11/1999 [↑](#endnote-ref-37)
38. <https://www.argentina.gob.ar/sites/default/files/res_cfe_361_19.pdf> [↑](#endnote-ref-38)
39. <https://publicholidays.com.bo/wp-content/uploads/2019/03/Bolivia_SchoolCalendar2019.png> [↑](#endnote-ref-39)
40. <https://www.justlanded.com/espanol/Brasil/Guia-Just-Landed/Educacion/La-vida-escolar-en-Brasil> [↑](#endnote-ref-40)
41. <https://www.leychile.cl/Navegar?idNorma=1019017#:~:text=Art%C3%ADculo%203%C2%BA%3A%20El%20a%C3%B1o%20lectivo,los%20que%20no%20lo%20est%C3%A9n> [↑](#endnote-ref-41)
42. <https://www.mineducacion.gov.co/1759/w3-article-364691.html?_noredirect=1> [↑](#endnote-ref-42)
43. <http://repositorio.inie.ucr.ac.cr/bitstream/123456789/311/1/06.04.01.1120.pdf> [↑](#endnote-ref-43)
44. <https://hoy.com.do/educacion-aprobo-el-calendario-escolar-2019-2020-y-esto-es-todo-lo-que-tienes-que-saber/> [↑](#endnote-ref-44)
45. <https://educarecuador.gob.ec/> [↑](#endnote-ref-45)
46. <https://historico.elsalvador.com/historico/134672/escuelas-publicas-pierden-el-35-de-los-dias-de-clase.html> [↑](#endnote-ref-46)
47. <https://www.prensalibre.com/guatemala/mineduc-adelanta-el-inicio-del-ciclo-escolar-para-subir-a-200-dias-de-clases/#:~:text=Los%20estudiantes%20deben%20recibir%20al,termin%C3%B3%20con%20193%20d%C3%ADas%20impartidos> [↑](#endnote-ref-47)
48. <https://www.elheraldo.hn/csp/mediapool/sites/ElHeraldo/Pais/story.csp?cid=581473&sid=299&fid=214> [↑](#endnote-ref-48)
49. <https://www.dof.gob.mx/nota_detalle.php?codigo=5439711&fecha=02/06/2016> [↑](#endnote-ref-49)
50. <https://www.laprensa.com.ni/2018/02/10/nacionales/2374955-ministerio-de-educacion-redujo-mas-el-calendario-escolar-para-el-2018> [↑](#endnote-ref-50)
51. <https://www.laestrella.com.pa/nacional/190923/ano-210-dias-cabo-2023#:~:text=comenzar%20a%20implementarlo%22.-,Panam%C3%A1%20tiene%20185%20d%C3%ADas%20de%20clases%20al%20a%C3%B1o.,condiciones%20del%20plantel%2C%20entre%20otros>. [↑](#endnote-ref-51)
52. <https://www.mec.gov.py/sigmec/resoluciones/6586-2019-PETTA.pdf> [↑](#endnote-ref-52)
53. <http://www.minedu.gob.pe/compromisos-gestion-escolar/pdf/norma-tecnica-anio-escolar-2018.pdf> [↑](#endnote-ref-53)
54. <https://www.elpais.com.uy/que-pasa/meta-dias-clase.html> [↑](#endnote-ref-54)
55. <https://www.aa.com.tr/es/mundo/desde-el-16-de-marzo-se-suspenden-clases-en-venezuela-debido-a-los-primeros-casos-de-covid-19-/1765688> [↑](#endnote-ref-55)
56. Data for Brasil, Dominican Republic, Guatemala, Honduras, Nicaragua, Panama, and Venezuela was not available and therefore results of internet distribution were estimated using the other countries in the sample with similar GDP and GDP growth in the last years 5. [↑](#endnote-ref-56)
57. Encuesta Permanente de Hogares-Modulo Conectividad 2019: https://www.indec.gob.ar/indec/web/Institucional-Indec-BasesDeDatos [↑](#endnote-ref-57)
58. Encuesta Nacional de Hogares 2019: https://www.ine.gob.bo/index.php/censos-y-banco-de-datos/censos/bases-de-datos-encuestas-sociales/ [↑](#endnote-ref-58)
59. CASEN 2017: http://observatorio.ministeriodesarrollosocial.gob.cl/casen-multidimensional/casen/basedatos.php [↑](#endnote-ref-59)
60. Gran Encuesta Integrada de Hogares 2020: http://microdatos.dane.gov.co/index.php/catalog/659/data_dictionary [↑](#endnote-ref-60)
61. Encuesta Nacional de Hogares 2019: http://sistemas.inec.cr/pad5/index.php/catalog/239/data-dictionary/F1?file_name=Enaho%202019 [↑](#endnote-ref-61)
62. Encuesta Nacional Multipropósito de Hogares 2019: https://www.ecuadorencifras.gob.ec/encuesta-nacional-multiproposito-de-hogares/ [↑](#endnote-ref-62)
63. Encuesta de Hogares de Propósitos Múltiples 2016: http://digestyc.microdatahub.com/index.php/catalog/17 [↑](#endnote-ref-63)
64. Encuesta Nacional de Ingresos y Gastos de los Hogares 2018: https://www.inegi.org.mx/programas/enigh/nc/2018/default.html#Microdatos [↑](#endnote-ref-64)
65. Encuesta Permanente de Hogares 2018: https://www.dgeec.gov.py/microdatos/microdatos.php [↑](#endnote-ref-65)
66. Encuesta Nacional de Hogares 2019: http://iinei.inei.gob.pe/microdatos/ [↑](#endnote-ref-66)
67. Encuesta Continua de Hogares 2019: http://www.ine.gub.uy/web/guest/encuesta-continua-de-hogares1 [↑](#endnote-ref-67)
68. <https://www.educ.ar/recursos/152610/programaci%C3%B3n-de-encuentro,-pakapaka-y-radio-escuela-en-seguimos-educando#gsc.tab=0> [↑](#endnote-ref-68)
69. https://www.educ.ar/recursos/151358/seguimos-educando-cuadernos?from=151381#gsc.tab=0 [↑](#endnote-ref-69)
70. https://www.minedu.gob.bo/index.php?option=com_content&view=article&id=4551:comienza-el-nuevo-diseno-de-la-educacion-en-bolivia&catid=182&Itemid=854 [↑](#endnote-ref-70)
71. https://socialdigital.iadb.org/es/covid-19/education/respuesta-regional/6078 [↑](#endnote-ref-71)
72. https://tab.uol.com.br/noticias/redacao/2020/06/16/educacao-a-distancia-e-o-futuro-pos-pandemia.htm?aff_source=56d95533a8284936a374e3a6da3d7996 [↑](#endnote-ref-72)
73. https://g1.globo.com/educacao/noticia/2020/07/06/60percent-dos-estados-monitoram-acesso-ao-ensino-remoto-resultados-mostram-apagao-do-ensino-publico-na-pandemia.ghtml [↑](#endnote-ref-73)
74. https://g1.globo.com/go/goias/noticia/2020/05/21/durante-pandemia-aulas-sao-dadas-virtualmente-mas-internet-limitada-e-dificuldade-de-manter-rotina-sao-desafios.ghtml [↑](#endnote-ref-74)
75. <https://www.mineduc.cl/apoyos-del-mineduc-durante-la-pandemia-del-covid-19/> [↑](#endnote-ref-75)
76. <https://en.unesco.org/fieldoffice/santiago/articles/minister-education-Chile-covid-19> [↑](#endnote-ref-76)
77. <https://www.mineducacion.gov.co/portal/salaprensa/Noticias/399326:Este-miercoles-en-Profe-en-tu-casa-aprendamos-sobre-criptografia-y-la-relacion-de-las-regularidades-con-las-matematicas> [↑](#endnote-ref-77)
78. <https://larepublica.pe/mundo/2020/05/04/aprendo-en-casa-costa-rica-como-y-donde-acceder-a-las-capacitaciones-y-clases-virtuales-durante-coronavirus-sinart-mep-uned-via-trece-cnt/> [↑](#endnote-ref-78)
79. <https://recursos.mep.go.cr/2020/aprendoencasa/> [↑](#endnote-ref-79)
80. <http://escuelasradiofonicas.edu.do/about-us-2/> [↑](#endnote-ref-80)
81. <https://educarecuador.gob.ec/> [↑](#endnote-ref-81)
82. <https://www.mined.gob.sv/emergenciacovid19/horarios.html> [↑](#endnote-ref-82)
83. <https://www.mined.gob.sv/jdownloads/Circulares%20Institucionales/Circulares%202020/Circular%20Ministerial%2010-2020.pdf> [↑](#endnote-ref-83)
84. <https://forbescentroamerica.com/2020/03/23/mineduc-difunde-guias-de-estudio-para-no-atrasar-ciclo-escolar/> [↑](#endnote-ref-84)
85. <https://www.se.gob.hn/detalle-articulo/1414/> [↑](#endnote-ref-85)
86. <https://www.aprendeencasa.mx/aprende-en-casa/acceso.html> [↑](#endnote-ref-86)
87. <http://www.meduca.gob.pa/sites/default/files/NORMATIVA%20OPERATIVA%20PARA%20EL%20APOYO%20EDUCATIVO%20MODALIDAD%20A%20DISTANCIA_compressed_1.pdf> [↑](#endnote-ref-87)
88. <http://www.meduca.gob.pa/sites/default/files/Resoluci%C3%B3n%20No.59%20Aprueba%20los%20lineamientos%20que%20organizan%20y%20orientan%20los%20procedimientos%20generales.pdf> [↑](#endnote-ref-88)
89. <https://www.mec.gov.py/cms_v2/adjuntos/15716?1589908264> [↑](#endnote-ref-89)
90. <https://aprendoencasa.pe/#/> [↑](#endnote-ref-90)
91. <https://www.ceibal.edu.uy/es> [↑](#endnote-ref-91)
92. <https://www.elespectador.com/noticias/educacion/uruguay-lleva-la-delantera-en-educacion-virtual-en-america-latina/> [↑](#endnote-ref-92)
93. <http://me.gob.ve/Descargas/PDF/PLANPEDAGOGICOMPPE15MAR.pdf> [↑](#endnote-ref-93)
